# Synthesis and Anticancer Activity of 2-(Alkyl-, Alkaryl-, Aryl-, Hetaryl-)-[1,2,4]triazolo[1,5-*c*]quinazolines

**DOI:** 10.3797/scipharm.1211-08

**Published:** 2012-12-23

**Authors:** Sergiy I. Kovalenko, Lyudmyla M. Antypenko, Andriy K. Bilyi, Sergiy V. Kholodnyak, Olexandr V. Karpenko, Olexii M. Antypenko, Natalya S. Mykhaylova, Tetyana I. Los, Olexandra S. Kolomoets

**Affiliations:** 1 Organic and Bioorganic Chemistry Department, Zaporizhzhya State Medical University, Mayakovsky Ave. 26, 69035, Zaporizhzhya, Ukraine.; 2 Enamine Ltd., Oleksandra Matrosova 23, Kyiv, 01103, Ukraine.

**Keywords:** [1,2,4]Triazolo[1,5-*c*]quinazolines, Anticancer activity, Carboxylic acid (3*H*-quinazoline-4-ylidene)hydrazides, *N-*(R-Benzylidene)-*N′-(*3*H*-quinazoline-4-ylidene)hydrazines, 2-Alkyl (alkaryl-, aryl-, hetaryl-)[1,2,4]triazolo[1,5-*c*]quinazolines

## Abstract

The combinatorial library of novel potential anticancer agents, namely, 2-(alkyl-, alkaryl-, aryl-, hetaryl-)[1,2,4]triazolo[1,5-*c*]quinazolines, was synthesized by the heterocyclization of the alkyl-, alkaryl-, aryl-, hetarylcarboxylic acid (3*H*-quinazoline-4-ylidene)hydrazides by oxidative heterocyclization of the 4-(arylidenehydrazino)quinazolines using bromine, and by the heterocyclization of *N*-(2-cyanophenyl)formimidic acid ethyl ester. The optimal method for synthesis of the *s*-triazolo[1,5-*c*]quinazolines appeared to be cyclocondensation of the corresponding carboxylic acid (3*H*-quinazoline-4-ylidene)hydrazides. The compounds’ structures were established by ^1^H, ^13^C NMR, LC- and EI-MS analysis. The *in vitro* screening of anticancer activity determined the most active compound to be 3,4,5-trimethoxy-*N*′-[quinazolin-4(3*H*)-ylidene]benzohydrazide (**3.20**) in micromolar concentrations with the GI_50_ level (MG_MID, GI_50_ is 2.29). Thus, the cancer cell lines whose growth is greatly inhibited by compound **3.20** are: non-small cell lung cancer (NCI-H522, GI_50_=0.34), CNS (SF-295, GI_50_=0.95), ovarian (OVCAR-3, GI_50_=0.33), prostate (PC-3, GI_50_=0.56), and breast cancer (MCF7, GI_50_=0.52), leukemia (K-562, GI_50_=0.41; SR, GI_50_=0.29), and melanoma (MDA-MB-435, GI_50_=0.31; SK-MEL-5, GI_50_=0.74; UACC-62, GI_50_=0.32). SAR-analysis is also discussed.

## Introduction

It is known, that [1,2,4]triazolo[1,5-*c*]quinazoline derivatives possess adenosine and benzodiazepine receptor affinity [1, 2], antiasthmatic, tranquilizing, neurostimulating [3], phosphodiesterase 10A inhibitive [4], antimicrobial and antifungal [5–7], anti-inflammatory, and sedative activities [8, 9]. As a result, their derivatives surely will have biological activity because of the potent pharmacophore they have in their structures. It is reported that more than half of the top 25 health problems are oncology indications [10]. Breast cancer comes out on top as the single most targeted disease. Upon analyzing the pharmaceutical market of 2011 year, among the 33 new active substances, there were eight anticancer drugs gaining their first approval in indication. And four of them, Crizotinib (Xalkori), Ruxolitinib (Jakafi), Vandetanib (Caprelsa), Vemurafenib (Zelboraf), are kinase inhibitors [10]. While investigation of [1,2,4]triazolo[1,5-*c*]quinazoline anticancer activity is very poor, quinazoline [11, 12] and 1,2,4-triazole [13–16] derivatives are already reported to be anticancer drugs or demonstrate antitumor properties as kinase inhibitors. Hence, the aim of our research was the formation of the new class of potent anticancer agents by fusion of the quinazoline and triazole ring and modification of their derivatives.

## Results and Discussion

The starting compounds **3.1**–**3.41** for the synthesis of the *s*-triazolo[1,5-*c*]quinazolines were obtained by the treatment of 4-chloroquinazoline (**1.1**) with acid hydrazides (method A) or by acylation of (3*H*-quinazoline-4-ylidene)hydrazine (**2.1**) with corresponding carboxylic acids, activated by *N,N*-carbonyldiimidazole (method B) in anhydrous dioxane (Scheme 1) [17, 18]. The hydrazones **4.1–4.6** were synthesized by the interaction of the (3*H*-quinazoline-4-ylidene)hydrazine (**2.1**) with aromatic aldehydes in 2-propanol or dioxane (Scheme 1) [18].

In the ^1^H NMR spectra, the low-field signals of the quinazoline endocyclic NH-group and amide group were observed at 11.99–9.48 ppm, which resonate together as a singlet (**3.1–3.3, 3.27, 3.36**) or multiplet (**3.12, 3.14, 3.17–3.20, 3.23–3.26, 3.29, 3.30, 3.36, 3.40, 3.41**). In some cases, these protons are doubled (**3.6, 3.7, 3.11, 3.13, 3.32, 3.33, 3.34, 3.38**) or do not even occur due to the rapid exchange of deuterium (**3.16, 3.21**). The aromatic protons of the quinazoline cycle that form the characteristic ABCD-system (doublets H-8 and H-5; triplets H-6 and H-7), were doubled (**3.1–3.3**) or resonate with aromatic protons as multiplets (**3.6, 3.7, 3.10–3.13, 3.20–3.25, 3.28–3.34, 3.36, 3.38, 3.39**). Proton H-2 appeared as a singlet at 8.14–7.64 ppm (**3.8, 3.12, 3.17, 3.27, 3.37, 3.40, 3.41**), or as a multiplet with other aromatic protons (**3.7, 3.10, 3.11, 3.13, 3.14, 3.16, 3.18–3.25, 3.28–3.34, 3.36, 3.38, 3.39**) or a doubled singlet at 8.08–8.04, 7.92–7.73 ppm (**3.1–3.3, 3.6, 3.26**). It is important to mention that the signals doubling the exchange protons, the quinazoline cycle protons, aromatic, and aliphatic part of **3.1–3.41,** occur due to the prototropic (hydrazino-hydrazone) tautomerism (ratio of tautomeric forms is 1:1 according to integral curves).

In the ^1^H NMR spectra of compounds **4.1–4.6,** there was a characteristic singlet of the azomethine proton at 8.80–8.30 ppm and a low-field broadened NH singlet of the quinazoline cycle at 11.20–15.60 ppm. In addition, the synthesized compounds were also characterized by the ABCD-system of the quinazoline cycle and substituents with appropriate multiplicity. The location of the azomethine group’s proton signals at 8.80–8.30 ppm provides an opportunity to affirm the *trans* arrangement of substituents around the double bond of the azomethine group [19].

Mass spectra of compounds **3.14, 3.15** were characterized by low intensity molecular ions [M]^+•^ and [M+1]^+^, the main fragmentation of which was associated with the formation of fragmentary ions [ArCO]^+•^ with *m/z* 119 (100%, **3.14**), 173 (90.1%, **3.15**), and ion [Ar]^+•^ with *m/z* 91 (38.9%, **3.14**) and 145 (44.9%, **3.15**). These spectra also showed the ion [HetN-NH]^+•^ with *m/z* 159, which had subsequently lost N_2_ and then fragmentized like a quinazolinium-cation.

Annelation of the triazole ring to the quinazoline cycle was also performed by heterocyclization of the alkyl (arylalkyl-, aryl-) carboxylic acid (3*H*-quinazolin-4-ylidene)-hydrazides (**3.1–3.40**) in glacial acetic acid or by oxidative heterocyclization of 4-arylidene-hydrazinoquinazolines (**4.1–4.6**) with bromine in glacial acetic acid (Scheme 1) [21]. In addition, compounds **5.7, 5.12, 5.17, 5.18, 5.23, 5.27, 5.31, 5.33** were obtained by the heterocyclization of *N*-(2-cyanophenyl)formimidic acid ethyl ester with aromatic acid hydrazides (Scheme 1). The latter method was less effective than the previous because of a lower yield. It is important, that in all cases, intermediate [1,2,4]triazolo[4,3-*c*]-quinazolines are ANRORC-rearranged forming 2-R-[1,2,4]triazolo[1,5-*c*]quinazolines (**5.1–5.40**) [21]. The ^1^H-NMR spectra of compounds **5.1–5.40** were significantly different from the spectra of hydrazides **3.1–3.40** and hydrazones **4.1–4.6**. Thus, the characteristic low-field singlet H-5 of the *s*-triazolo[1,5-*c*]quinazolines ring resonated at 9.85–9.25 ppm. It is important that the chemical shift of this proton was associated with the donor-acceptor properties of the substituent at position 2. In the case of pronounced donor properties of the substituents (**5.1–5.4, 5.7, 5.11, 5.14, 5.20**), the H-5 singlet was shown at 9.36–9.25 ppm, while the acceptor substituents displaced it in the low-field part of the spectrum (9.85–9.60 ppm). Other protons of the quinazoline cycle were registered at 8.56–7.71 ppm as consecutive doublets (H-10 and H-7) and triplets (H-8 and H-9). In addition, compounds **5.1–5.40** were characterized by corresponding multiplicity and chemical shifts of the functional groups at position 2 of the protons signals.

The signals of carbon at the 2 and 5 positions in the ^13^C NMR spectra were characteristic for triazolo[1,5-*c*]quinazolines (compounds **5.2**, **5.4**, **5.12**, **5.17**, **5.24**, **5.32**, **5.34**, **5.37**, **5.38**). The carbon in position 2 was electron-deficient and registered in the low-field at 159.47–164.40 ppm. At the same time, the carbon in position 5, even though it is located between two nitrogen atoms, was less deshielded and was found at 134.32–139.69 ppm.

In the LC-MS spectra, compounds **5.1–5.40** were characterized by positive ions [M+1]. The spectra of compounds **3.23–3.27** with chlorine atoms in the molecule have additional molecular ion [M+3]. The mass spectra (EI) of the 2-R-[1,2,4]triazolo[1,5-*c*]quinazolines (**5.1, 5.2, 5.7, 5.9, 5.12, 5.14, 5.15, 5.18, 5.19, 5.22, 5.26, 5.27, 5.30–5.38**) showed the aromaticity of the tricyclic system: the molecular peak M^+•^ for the majority of compounds had the maximum intensity. The molecular peak of these compounds was characterized by fragmentation of the main line of the C(10b)-N(1) and N(3)-N(4) with cleavage of the amidine fragment ([RC(N)N]^+^) and by the formation of the ion whose mass corresponded to the calculated quinazoline mass (*m/z* 129). In the latter case, the destruction of the triazole cycle occurred. The further expansion pattern was typical for the quinazolinium-cation.

Some of the synthesized compounds had peculiarities of fragmentation under electron impact. Thus, the spectrum of compound **5.1** had two equivalent directions of [M]^+•^ fragmentation, firstly, by the N(1)-C(2) and N(3)-N(4) bonds with the formation of the ion [M-C_2_H_3_N]^+^ with *m/z* 143, and secondly, classically, by the C(10b)-N(1) and N(3)-N(4) bonds with the formation of [CH_3_C(N)N]^+^ and the quinazolinium-cation (*m/z* 129). This fact indicates that the methyl group in the position 2 destabilized the heterocyclic aromaticity.

Compound **5.18** also had a specific fragmentation, notably the previous emission of the molecular ion radicals CH_3_ (*m/z* 262), -OCH_3_ (*m/z* 245), and HCN (*m/z* 248), which was probably due to the *o*-effect of the substituent that changed the distribution of electron density in the triazoloquinazoline ring. Compound **5.9** was characterized by a high-intensity ion with *m/z* 91 (100%), because of the classic “benzyl collapse”, which led to the formation of the stable tropylium ion. The mass spectra of compounds **5.26** and **5.27** had two peaks with equal intensity (*m/z* 324 (100–86.2%), *m/z* 326 (93.5–87.9%), which confirms the presence of bromine atoms.

The interaction of (3*H*-quinazoline-4-ylidene)hydrazine (**2.1**) with cyclic anhydrides of dicarboxylic acids (succinic and phthalic anhydrides) in propan-2-ol or dioxane led to the ring opening and formation of the corresponding *N*-acyl derivatives **6.1, 6.3** (Scheme 2) [22]. The monoethyl esters of the oxalic acid 2-((3*H*)-quinazoline-4-ylidene)hydrazide (**13.1**) were synthesized by the treatment of **2.1** with chloroethyloxalate in the presence of triethylamine (Scheme 2). *s*-Triazolo[1,5-*c*]quinazolines (**7.1, 7.2**) were formed by cyclo-condensation of *N*-acyl derivatives **6.1** and **6.2** with proper anhydrides, as well as 2-[quinazolin-4(3*H*)-ylideneamino]-1*H*-isoindole-1,3(2*H*)-dione (**7.3**) was obtained from compound **6.3**.

The ^1^H-NMR spectra of compounds **7.1** and **7.2** were characterized by the low-field singlet H-5, which resonated at 9.52 and 9.25 ppm respectively, protons of the quinazoline cycle at 8.54-7.71 ppm were found as consecutive doublets (H-10 and H-7), and triplets (H-8 and H-9). The ^13^C NMR spectrum of compound **7.1** was characterized by the signals of the carbon atoms in position 2 and 5 at 159.92 ppm and 139.69 ppm. The sp^2^ hybridized carbon atom of the CO-group of **7.1** was observed in the low-fields at 156.05 ppm. The mass spectrum (EI) of [1,2,4] triazolo[1,5-*c*]quinazoline-2-ylpropionic acid (**7.2**) was characterized by a low intensity peak of the molecular ion M^+•^, due to the presence of a carboxyl group. The primary process of the **7.2** M^+•^ fragmentation was specified by the elimination of the OH and COOH radicals ([M-OH]^+•^ is 9.5% and [M-COOH]^+^ is 100%). Further decay patterns coincided with the previously given 2-R-[1,2,4]triazolo[1,5-*c*]-quinazolines destruction direction, i.e. there was destruction of the 1,2,4-triazole ring by the C(10b)-N(1) and N(3)-N(4) bonds, followed by fragmentation of the quinazoline bicycle.

In the ^1^H-NMR spectra of compound **7.3**, a two-proton multiplet was observed at 8.39 ppm, which characterized the proton at position 2 and 5 of the quinazoline cycle. Other aromatic protons of compound **7.3** formed a broad multiplet (8.20–7.60 ppm) with an intensity of seven proton units.

The ^13^C NMR spectrum of compound **7.3** was characterized by the equivalent signals of the unshielded carbon atoms in position 1 and 3 of the izoindol cycle at 166.18 ppm. The mass spectrum (EI) of the latter compound was characterized by the molecular ion (M^+•^, *m/z* 290, 49.5%), whose main ways of destruction were related to the elimination of particles CO and OH and formed the fragmented ion with *m/z* 245 (100%).

### Pharmacology

#### Anticancer assay for preliminary in vitro testing

Synthesized (3*H*-quinazolin-4-yliden)hydrazide carboxylic acids (**3**), dicarboxylic acid (3*H*-quinazolin-4-ylidene)hydrazides (**6**), *N-*(R-benzyliden)-*N′-*(3*H*-quinazolin-4-ylidene)-hydrazines (**4**), 2-R-[1,2,4]triazolo[1,5-c]quinazolines (**5**), ([1,2,4]triazolo[1,5-*c*]quinazolin-2-yl)carboxylic acids (**7.1**, **7.2**), and 2-[quinazolin-4(3*H*)-ylidenamino]-1*H*-isoindole-1,3(2*H*)-dione (**7.3**) were evaluated for antitumor activity against 60 cancer lines in a concentration of 10 μM. The human tumor cell lines were derived from nine different cancer types: leukemia, melanoma, lung, colon, CNS, ovarian, renal, prostate, and breast cancers. Primary anticancer assays were performed according to the US NCI protocol [23–25]. The compounds were added at a single concentration and the cell culture was incubated for 48 h. End point determinations were made with a protein binding dye, sulforhodamine B (SRB). The results for each compound are reported as the percent growth of treated cells when compared to untreated control cells (Table 1). It appears that individual cell lines have different sensitivities to the synthesized compounds.

Most of all carboxylic acid hydrazides **3** showed selective cytotoxic activity to cell lines of the NSC lung cancer (**3.10, 3.20, 3.23, 3.28, 3.29**) and breast cancer (**3.10, 3.20, 3.23, 3.26, 3.28, 3.29**). Hydrazones (**4**) also inhibited the growth of ovarian cancer cell lines by 35.2–44.0%. While hydrazides of dicarboxylic acids (**6**) are practically inactive compounds, they exhibited a high activity only against the cell line IGROV1 of ovarian cancer (**6.2**) and an antiproliferative effect against the cell line HS 578T of breast cancer (**6.1** and **6.3**).

Among all substances, 3,4,5-trimethoxy-*N*′-[quinazolin-4(3*H*)-ylidene]benzohydrazide (**3.20**) showed the widest range of the anticancer activity. This compound inhibited the growth of 55 cancer cell lines, namely NSC lung (A549/ATCC, HOP-62, NCI-H23, NCI-H460, NCI-H522), colon (HCT-116, HCT-15, HT29, KM12, SW-620), breast (MCF7), ovarian (OVCAR-3, OVCAR-8), renal (786-0, ACHN, CAKI-1, RXF 393), CNS (SF-539, SNB-19, SNB-75) cancers, leukemia (CCRF-CEM, K-562, MOLT-4, RPMI-8226, SR), and melanoma (MALME-3M, UACC-257). Compound **3.20** showed an antiproliferative effect against the cell lines of NSC lung (HOP-92), breast (HS 578T, MDA-MB-435), renal (A498), CNS cancer (SF-295), leukemia (HL-60 (TB), and melanoma (SK-MEL-5).

In most cases, 2-R-[1,2,4]triazolo[1,5-*c*]quinazolines (**5**) did not show significant antitumor activity. Noteworthy compounds were **5.4**, **5.5,** and **5.7,** which had an antiproliferative effect against the HS 578T breast cancer cell line. The latter substances had chloromethylene (**5.4**), 2,4,5-trichlorophenoxymethylene (**5.5**), and benzyl (**5.7**) substituents in position 2. Inserting the phenylvinylic substituent (**5.10**) in position 2 led to a widening of the anticancer activity range (40 cell lines). Thus, compound **5.10** showed pronounced cytotoxicity against the cell lines of NSC lung (HOP-92, NCI-H460), breast (NCI/ADR-RES), ovarian (OVCAR-8), renal (RXF 393), CNS (SF-295) cancer, leukemia (SR), and melanoma (SK-MEL-5), and an antiproliferative effect against the cell lines of breast cancer (MDA-MB-435). The addition of the aryl substituent to position 2 did not affect the antitumor activity. But compounds with phenyl- (**5.12**), 2-hydroxyphenyl- (**5.16**), 4-methoxyphenyl- (**5.19**), and 4-nitrophenyl- (**5.29**) groups in the latter position were toxic to cancer cells, as well as the introduction of the hetaryl substituents, namely, furyl- (**5.31**), 2-benzofuryl- (**5.36**), 3-substituted pyrrole- (**5.35**), and 2-indole (**5.38**).

#### Through dose-dependent study in vitro on 60 cancer cell lines

Compounds **3.20** and **5.10** were chosen by the NCI for dose-dependent action in five concentrations according to a standard procedure in 58 cell lines of nine types of cancer (100μM–0.01μM), and was investigated [21–23]. Three dose-dependent parameters were calculated: 1) GI_50_ – molar concentration of the compound that inhibits 50% of net cell growth; 2) TGI – molar concentration of the compound leading to the total inhibition of cell growth; 3) LC_50_ – molar concentration of the compound leading to 50% net cell death. Furthermore, the mean graph midpoints (MG_MID) were calculated for each of the parameters, giving an average activity parameter over all of the cell lines for tested compounds. For the calculation of the MG_MID, insensitive cell lines are included with the highest tested concentration (Table 2).

The data in Table 2 show, that 4,5-trimethoxy-*N*′-[quinazolin-4(3*H*)-ylidene]benzohydrazide (**3.20**) has high cytotoxicity to all cancer cell lines, namely, NSC lung cancer (NCI-H522, GI_50_=0.34), CNS (SF-295, GI_50_=0.95), ovarian (OVCAR-3, GI_50_=0.33), prostate (PC-3, GI_50_=0.56) and breast (MCF7, GI_50_=0.52) cancer, leukemia (K-562, GI_50_=0.41; SR, GI_50_=0.29) and melanoma (MDA-MB-435, GI_50_=0.31; SK-MEL-5, GI_50_=0.74; UACC-62, GI_50_=0.32). Compound **5.10** is less active and negatively effected colon (HCT-15, GI_50_ = 0.89), breast (MDA-MB-231/ATCC, GI_50_ = 0.34) cancer and melanoma (MDA-MB-435, GI_50_ = 0.71). However, compound **5.10** possesses the most pronounced antitumor impact against the NSC lung cancer cell line HOP-62 (GI_50_ = 0.03).

Hence, the combinatorial library of novel alkyl(arylalkyl-, aryl-) carboxylic acid (3*H*-quinazolin-4-ylidene)hydrazides, *N-*(R-benzyliden)-*N-(*3*H*-quinazolin-4-ylidene)hydrazines, and 2-alkyl(alkaryl-, aryl-, hetaryl-)-[1,2,4]triazolo[1,5-*c*]quinazolines as promising anti-cancer agents was developed. The anticancer screening has determined that the above classes of compounds exhibited significant anticancer activity against several cancer cell lines.

## Conclusion

In the present paper, the combinatorial library of novel promising anticancer carboxylic acid (3*H*-quinazolin-4-yliden)hydrazides, *N-*(R-benzyliden)-*N-(*3*H*-quinazolin-4-ylidene)-hydrazines, and 2-alkyl(alkaryl-, aryl-, hetaryl-)-[1,2,4]triazolo[1,5-*c*]quinazolines was described. Forty-three synthesized compounds were tested for antitumor activity against leukemia, melanoma, lung, colon, CNS, ovarian, renal, prostate, and breast cancer cell lines. This investigation found that the most active compound was 3,4,5-trimethoxy-*N*′- (quinazolin-4(3*H*)-ylidene)benzohydrazide (**3.20**), which had 2.29 MG_MID GI_50_ in micromolar concentration. The cell lines of NSC lung cancer (NCI-H522, GI_50_=0.34), CNS (SF-295, GI_50_=0.95), ovarian (OVCAR-3, GI_50_=0.33), prostate (PC-3, GI_50_=0.56), and breast (MCF7, GI_50_=0.52) cancers, leukemia (K-562, GI_50_=0.41; SR, GI_50_=0.29), and melanoma (MDA-MB-435, GI_50_=0.31; SK-MEL-5, GI_50_=0.74; UACC-62, GI_50_=0.32) showed the highest sensitivity to compound **3.20**. SAR results could be used for further purposeful optimization of the leading compounds in the more effective anticancer agents aiming the next phase of target investigation.

## Experimental

### Chemistry

Melting points were determined in open capillary tubes and were uncorrected. The elemental analyses (C, H, N, S) were performed using the ELEMENTAR Vario EL Cube Analyzer (USA). Analyses were indicated by the symbols of the elements or functions within ±0.3% of the theoretical values. The ^1^H NMR spectra (400 MHz) and ^13^C NMR spectra (125 MHz) were recorded on the Varian-Mercury 400 (Varian Inc., Palo Alto, CA, USA) spectrometers with TMS as the internal standard in DMSO-*d_6_* solution. LC-MS were recorded using a chromatography / mass spectrometric system which consisted of the high-performance liquid chromatograph «Agilent 1100 Series» (Agilent, Palo Alto, CA, USA) equipped with a diode-matrix and mass-selective detector «Agilent LC/MSD SL» (atmospheric pressure chemical ionization – APCI). The electron impact mass spectra (EI-MS) were recorded on the Varian 1200 L instrument at 70 eV (Varian, USA). The purity of all obtained compounds was checked by ^1^H-NMR and LC-MS.

4-Chloroquinazolines (**1.1**) and 4-hydrazinoquinazolines (**2.1**) were synthesized according to the reported procedures [26]. Other starting materials and solvents were obtained from commercially available sources and used without additional purification.

### General procedure for (3H-quinazolin-4-ylidene)carbohydrazides (3.1–3.41)

*Method A.* To a solution of 1.65 g (10 mM) of 4-chloroquinazoline (**1.1**) in 10 ml of dioxane, the corresponding carboxylic acid hydrazide (11 mM) was added and kept in a water bath at 60°C for 8 h. After cooling, the reaction mixture was poured into the water, and a 5% solution of sodium bicarbonate was added to achieve pH 6–7. The formed precipitate was filtered off and dried.

*Method B.* To a solution of the corresponding carboxylic acid (11 mM) in 10 ml of anhydrous dioxane, 1.95 g (11 mM) of carbonyldiimidazole was added and heated in a water bath at 60–80°C for 1 hour, under the calcium chloride tube. While stirring, 4-hydrazinoquinazoline (**1.1**) was added to the reaction mixture 1.60 g (10 mM) and left for 8 h at room temperature. The mixture was poured into the water and acetic acid was added to achieve pH 6–7. The formed precipitate was filtered off and dried.

#### N′-[Quinazolin-4(3H)-ylidene]acetohydrazide (**3.1**)

Yield: Method A, 82%; Method B, 62%; M.p. 217–219°C (H_2_O); ^1^H-NMR (400 MHz) δ: 9.48 (s, 2H, NH), 8.11/7.87 (d, 1H, *J* = 8.0, H-5), 8.07/7.74 (s, 1H, H-2), 7.53/7.36 (t, 1H, *J*=7.8, H-7), 7.41/7.12 (d, 1H, *J* = 8.0, H-8), 7.32/7.18 (t, *J*=7.8, 1H, H-6), 2.05/2.23 (s, 3H, C*H*_3_); LC-MS, *m/z* = 203 [M+1]; Anal. calcd. for C_10_H_10_N_4_O: C, 59.40; H, 4.98; N, 27.71; Found: C, 59.38; H, 4.97; N, 27.70.

#### N′-[Quinazolin-4(3H)-ylidene]propanehydrazide (**3.2**)

Yield: Method A, 67%; Method B, 70%; M.p. 184–186°C (H_2_O); ^1^H-NMR (400 MHz) δ: 9.48 (s, 2H, NH), 8.09/7.86 (d, 1H, *J* = 8.0, H-5), 8.04/7.73 (s, 1H, H-2), 7.50/7.34 (t, 1H, *J*=7.9, H-7), 7.38/7.11 (d, *J*=7.9, 1H, H-8), 7.28/7.16 (t, 1H, *J*=8.0, H-6), 2.65/2.30 (q, 2H, *J*=7.0, C*H*_2_CH_3_), 1.17 (m, 3H, CH_2_C*H*_3_); LC-MS, *m/z* = 217 [M+1]; Anal. calcd. for C_11_H_12_N_4_O: C, 61.10; H, 5.59; N, 25.91; Found: C, 61.08; H, 5.57; N, 25.90.

#### N′-[Quinazolin-4(3H)-ylidene]butanehydrazide (**3.3**)

Yield: Method A, 66%; Method B, 59%; M.p. 104–106°C (H_2_O); ^1^H-NMR (400 MHz) δ: 9.48 (s, 2H, NH), 8.08/7.86 (d, 1H, *J* = 8.0, H-5), 8.02/7.71 (s, 1H, H-2), 7.49/7.34 (t, 1H, *J* = 7.7, H-7), 7.37/7.11 (d, 1H, *J* = 8.1, H-8), 7.28/7.18 (t, 1H, *J* = 7.8, H-6), 2.64/2.28 (t, 2H, *J* = 7.1, CO-CH_2_), 1.69 (m, 2H,*C*H_2_CH_3_), 1.02 (m, 3H, CH_3_); LC-MS, *m/z* = 231 [M+1]; Anal. calcd. for C_12_H_14_N_4_O: C, 62.59; H, 6.13; N, 24.33; Found: C, 62.58; H, 6.14; N, 24.30.

#### 2-Chloro-N′-[quinazolin-4(3H)-ylidene]acetohydrazide (**3.4**)

Yield: Method B, 76%; M.p. 189–191°C (DMF–H_2_O); LC-MS, *m/z* = 237 [M+1], 239 [M+3]; Anal. calcd. for C_10_H_9_ClN_4_O: C, 50.75; H, 3.83; Cl, 14.98; N, 23.67; Found: C, 50.78; H, 3.84; Cl, 14.99; N, 23.68.

#### N′-[Quinazolin-4(3H)-ylidene]-2-(2,4,5-trichlorophenoxy)acetohydrazide (**3.5**)

Yield: Method B, 100%; M.p. 272–274°C (DMF–H_2_O); LC-MS, *m/z* = 397 [M], 399 [M+2], 401 [M+4], 403 [M+6]; Anal. calcd. for C_16_H_11_Cl_3_N_4_O_2_: C, 48.33; H, 2.79; Cl, 26.75; N, 14.09; Found: C, 48.32; H, 2.78; Cl, 26.73; N, 14.08.

#### 2-Oxo-2-[2-(quinazolin-4(3H)-ylidene)hydrazinyl]acetonitrile (**3.6**)

Yield: Method B, 89%; M.p. 212–214°C (dioxane); ^1^H-NMR (400 MHz) δ: 11.73 (s, 1H, NH), 10.72/9.95 (s, 1H, NH), 7.99/7.89 (m, 1H, H-5), 7.58/7.45 (m, 1H, H-2), 7.38/7.16 (m, 3H, H-7, H-8, H-6), 4.16/3.88 (s, 2H, -CH_2_); LC-MS, *m/z* = 226 [M-1]; Anal. calcd. for C_10_H_7_N_5_O: C, 56.34; H, 3.31; N, 32.85; Found: C, 56.35; H, 3.33; N, 32.88.

#### 2-Phenyl-N′-[quinazolin-4(3H)-ylidene]acetohydrazide (**3.7**)

Yield: Method A, 94%; Method B, 87%; M.p. 182–184°C (ethanol–H_2_O); ^1^H-NMR (400 MHz) δ: 11.61 (s, 1H, NH), 10.38/9.65 (s, 1H, NH), 8.2-7.2 (m, 10H, H_arom_), 4.00/3.63 (s, 2H, C*H*_2_Ph); LC-MS, *m/z* = 279 [M+1]; Anal. calcd. for C_16_H_14_N_4_O: C, 69.05; H, 5.07; N, 20.13; Found: C, 69.03; H, 5.03; N, 20.12.

#### 2-Hydroxy-2,2-diphenyl-N′-[quinazolin-4(3H)-ylidene]acetohydrazide (**3.8**)

Yield: Method B, 82%; M.p. 270–272°C (DMF–H_2_O); ^1^H-NMR (400 MHz) δ: 11.45 (s, 1H, NH), 11.24 (s, 1H, NH), 8.02 (d, 1H, *J* = 7.8, H-5), 7.83 (s, 1H, H-2), 7.36 (t, 1H, *J* = 7.8, H-7), 7.62-7.08 (m, 12H, H_arom_), 6.61 (s, 1H, OH); LC-MS, *m/z* = 371 [M+1]; Anal. calcd. for C_22_H_18_N_4_O_2_: C, 71.34; H, 4.90; N, 15.13; Found: C, 71.35; H, 4.93; N, 15.12.

#### 3-Phenyl-N′-[quinazolin-4(3H)-ylidene]propanehydrazide (**3.9**)

Yield: Method A, 96%; Method B, 96%; M.p. 180–182°C (ethanol–H_2_O); LC-MS, *m/z* = 293 [M+1]; Anal. calcd. for C_17_H_16_N_4_O: C, 69.85; H, 5.52; N, 19.17; Found: C, 69.85; H, 5.53; N, 19.17.

#### (2E)-3-Phenyl-N′-[quinazolin-4(3H)-ylidene]acrylohydrazide (**3.10**)

Yield: Method B, 78%; M.p. 218–220°C (DMF–H_2_O); ^1^H-NMR (400 MHz) δ: 11.67 (s, 1H, NH), 10.63 (s, 1H, NH), 7.92-7.16 (m, 12H, H_arom_); LC-MS, *m/z* = 291 [M+1]; Anal. calcd. for C_17_H_14_N_4_O: C, 70.39; H, 4.86; N, 19.30; Found: C, 70.37; H, 4.83; N, 19.32.

#### 2-(1H-Indol-3-yl)-N′-[quinazolin-4(3H)-ylidene]acetohydrazide (**3.11**)

Yield: Method B, 75%; M.p. 230–232°C (DMF–H_2_O); ^1^H-NMR (400 MHz) δ: 11.52/10.09 (s, 1H, NH), 10.24/9.55 (s, 1H, NH), 8.0–6.9 (m, 10H, H_arom_), 4.05/3.71 (s, 2H, CH_2_); LC-MS, *m/z* = 320 [M+3]; Anal. calcd. for C_18_H_15_N_5_O: C, 68.13; H, 4.76; N, 22.07; Found: C, 68.12; H, 4.73; N, 22.08.

#### N′-[Quinazolin-4(3H)-ylidene]benzohydrazide (**3.12**)

Yield: Method A, 87%; Method B, 91%; M.p. 240–242°C (dioxane–H_2_O); ^1^H-NMR (400 MHz) δ: 10.56 (m, 2H, NH), 8.21 (d, 1H, *J*=8.0, H-5), 8.05 (s, 1H, H-2), 7.89 (d, 2H, H-2′,6′), 7.5–7.3 (m, 6H, H-3′, H-4′, H-5′,H-6, H-7, H-8); LC-MS, *m/z* = 265 [M+1], 266 [M+2]; Anal. calcd. for C_15_H_12_N_4_O: C, 68.17; H, 4.58; N, 21.20; Found: C, 68.15; H, 4.57; N, 21.21.

#### 3-Methyl-N′-[quinazolin-4(3H)-ylidene]benzohydrazide (**3.13**)

Yield: Method B, 85%; M.p. 256–258°C (dioxane-H_2_O); ^1^H-NMR (400 MHz) δ: 11.77 (s, NH), 10.63/10.33 (s, NH), 8.62-7.14 (m, 9H, H_arom_); LC-MS, *m/z* = 279 [M+1], 280 [M+2]; Anal. calcd. for C_16_H_14_N_4_O: C, 69.05; H, 5.07; N, 20.13; Found: C, 69.07; H, 5.09; N, 20.12.

#### 4-Methyl-N′-[(quinazolin-4(3H)-ylidene]benzohydrazide (**3.14**)

Yield: Method A, 85%; M.p. 268–270°C (dioxane-H_2_O); ^1^H-NMR (400 MHz) δ: 10.65 (m, 2H, NH), 8.6-7.3 (m, 9H, H_arom_), 2.50 (s, 1H, C*H*_3_); EI-MS, *m/z* (I_rel_, %) = 279(1.1), 278 (M^+•^, 6.7), 159(4.7), 145(4.2), 129 (1.7), 120(7.8), 119(100), 103(4.6), 91(38.9), 65(8.8); LC-MS, *m/z* = 279 [M+1]; Anal. calcd. for C_16_H_14_N_4_O: C, 69.05; H, 5.07; N, 20.13; Found: C, 69.07; H, 5.09; N, 20.15.

#### N′-[Quinazolin-4(3H)-ylidene]-4-(trifluoromethyl)benzohydrazide (**3.15**)

Yield: Method A, 84%; Method B, 86%; M.p. 252–254°C (dioxane-H_2_O); EI-MS, *m/z* (I_rel_, %) = 333 (7.0), 332 (M^+•^, 38.4), 174 (7.6), 173 (90.1), 160 (9.4), 159 (100), 145 (44.9), 130 (5.3), 129 (5.4), 104 (7.6), 103 (14.3), 102 (8.7), 95 (5.3), 77 (5.7), 76 (6.5), 75 (6.6); LC-MS, *m/z* = 333 [M+1]; Anal. calcd. for C_16_H_11_F_3_N_4_O: C, 57.83; H, 3.34; F, 17.15; N, 16.86; Found: C, 57.85; H, 3.36; F, 17.16; N, 16.88.

#### 2-Hydroxy-N′-[quinazolin-4(3H)-ylidene]benzohydrazide (**3.16**)

Yield: Method A, 96%; Method B, 96%; M.p. 298–300°C (dioxane-H_2_O); ^1^H-NMR (400 MHz) δ: 9.48 (s, 1H, NH), 8.09 (m, 2H, H-5, H-2), 7.95 (s, 1H, OH), 7.84 (d, 1H, *J* = 7.8, H-6′), 7.56 (t, 1H, *J* = 7.8, H-7), 7.38 (m, 1H, H-8, H-6), 7.14 (t, 1H, *J* = 7.8, H-4′), 6.62 (t, 1H, *J* = 7.8, H-5′), 6.88 (m, 1H, H-3′); LC-MS, *m/z* = 281 [M+1]; Anal. calcd. for C_15_H_12_N_4_O_2_: C, 64.28; H, 4.32; N, 19.99; Found: C, 64.29; H, 4.33; N, 20.01.

#### 4-Hydroxy-N′-[quinazolin-4(3H)-ylidene]benzohydrazide (**3.17**)

Yield: Method A, 86%; M.p. 247–249°C (dioxane-H_2_O); ^1^H-NMR (400 MHz) δ: 10.36 (m, 2H, NH), 9.63 (s, 1H, OH), 8.19 (d, 1H, *J*=8.0, H-5), 8.07 (s, 1H, H-2), 7.79 (d, 2H, *J*=7.8, H-2′, H-6′), 7.56 (t, 1H, *J*=7.9, H-7), 7.36 (m, 1H, H-6, H-8), 6.81 (d, 2H, *J*=7.8, H-3′, H-5′); LC-MS, *m/z* = 281 [M+1]; Anal. calcd. for C_15_H_12_N_4_O_2_: C, 64.28; H, 4.32; N, 19.99; Found: C, 64.29; H, 4.33; N, 20.01.

#### 2-Methoxy-N′-[quinazolin-4(3H)-ylidene]benzohydrazide (**3.18**)

Yield: Method A, 86%; M.p. 242–244°C (dioxane-H_2_O); ^1^H-NMR (400 MHz) δ: 11.99 (m, 2H, NH), 8.06 (m, 2H, H-2, H-6′), 7.99 (d, 1H, *J*=7.6, H-5), 7.52 (m, 2H, H-4′, H-5′), 7.33 (t, 1H, *J*=7.6, H-6), 7.23 (d, 1H, *J*=7.6, H-8), 7.20 (d, 1H, *J*=8.1, H-3′), 7.10 (t, 1H, *J*=7.6, H-7), 4.03 (s, 3H, OC*H*_3_); LC-MS, *m/z* = 295 [M+1]; Anal. calcd. for C_16_H_14_N_4_O_2_: C, 65.30; H, 4.79; N, 19.04; Found: C, 65.29; H, 4.76; N, 19.06.

#### 4-Methoxy-N′-[quinazolin-4(3H)-ylidene]benzohydrazide (**3.19**)

Yield: Method A, 93%; Method B, 92%; M.p. 220–222°C (dioxane-H_2_O); ^1^H-NMR (400 MHz) δ: 10.62 (m, 2H, NH), 8.21 (m, 2H, H-2, H-5), 7.88 (d, 2H, *J*=8.3, H-2′, H-6′), 7.69 (t, 1H, H-6), 7.51 (m, 2H, H-7, H-8), 7.05 (d, 2H, *J*=8.3, H-3′, H-5′), 3.82 (s, 3H, OC*H*_3_); LC-MS, *m/z* = 294 [M]; Anal. calcd. for C_16_H_14_N_4_O_2_: C, 65.30; H, 4.79; N, 19.04; Found: C, 65.28; H, 4.76; N, 19.02.

#### 3,4,5-Trimethoxy-N′-[quinazolin-4(3H)-ylidene]benzohydrazide (**3.20**)

Yield: Method B, 96%; M.p. 242–244°C (DMF–H_2_O); ^1^H-NMR (400 MHz) δ: 10.65 (m, 2H, NH), 8.6-7.1 (m, 7H, H_arom_), 3.88 (s, 6H, 3,5-(OCH_3_)_2_), 3.70 (s, 3H, 4-OCH_3_); LC-MS, *m/z* = 355 [M+1]; Anal. calcd. for C_18_H_18_N_4_O_4_: C, 61.01; H, 5.12; N, 15.81; Found: C, 61.02; H, 5.14; N, 15.82.

#### 2-Fluoro-N′-[quinazolin-4(3H)-ylidene]benzohydrazide (**3.21**)

Yield: Method B, 85%; M.p. 259–260°C (dioxane-H_2_O); ^1^H-NMR (400 MHz) δ: 10.98 (s, 1H, NH), 8.1-7.1 (m, 9H, H_arom_); LC-MS, *m/z* = 283 [M+1]; Anal. calcd. for C_15_H_11_FN_4_O: C, 63.83; H, 3.93; F, 6.73; N, 19.85; Found: C, 63.82; H, 3.90; F, 6.71; N, 19.82.

#### 4-Fluoro-N′-[quinazolin-4(3H)-ylidene]benzohydrazide (**3.22**)

Yield: Method A, 93%; Method B, 72%; M.p. 248–252°C (dioxane-H_2_O); ^1^H-NMR (400 MHz) δ: 11.72 (s, 1H, NH), 10.72 (s, 1H, NH), 8.58-7.1 (m, 9H, H_arom_); LC-MS, *m/z* = 283 [M+1], 284 [M+2]; Anal. calcd. for C_15_H_11_FN_4_O: C, 63.83; H, 3.93; F, 6.73; N, 19.85; Found: C, 63.85; H, 3.96; F, 6.75; N, 19.86.

#### 2-Chloro-N′-[quinazolin-4(3H)-ylidene]benzohydrazide (**3.23**)

Yield: Method A, 91%; M.p. 253–255°C (dioxane); ^1^H-NMR (400 MHz) δ: 10.51 (m, 2H, NH), 8.2-7.1 (m, 9H, H_arom_); LC-MS, *m/z* = 299 [M+1], 301 [M+3]; Anal. calcd. for C_15_H_11_ClN_4_O: C, 60.31; H, 3.71; Cl, 11.87; N, 18.76; Found: C, 60.34; H, 3.73; Cl, 11.88; N, 18.78.

#### 3-Chloro-N′-[quinazolin-4(3H)-ylidene]benzohydrazide (**3.24**)

Yield: Method B, 90%; M.p. 262–264°C (dioxane); ^1^H-NMR (400 MHz) δ: 10.80 (m, 2H, NH), 8.6-7.1 (m, 9H, H_arom_); LC-MS, *m/z* = 299 [M+1], 301 [M+3]; Anal. calcd. for C_15_H_11_ClN_4_O: C, 60.31; H, 3.71; Cl, 11.87; N, 18.76; Found: C, 60.30; H, 3.69; Cl, 11.84; N, 18.74.

#### 4-Chloro-N′-[quinazolin-4(3H)-ylidene]benzohydrazide (**3.25**)

Yield: Method A, 100%; M.p. 266–268°C (dioxane); ^1^H-NMR (400 MHz) δ: 10.85 (m, 2H, NH), 8.62–7.10 (m, 9H, H_arom_); LC-MS, *m/z* = 299 [M+1], 301 [M+3]; Anal. calcd. for C_15_H_11_ClN_4_O: C, 60.31; H, 3.71; Cl, 11.87; N, 18.76; Found: C, 60.33; H, 3.74; Cl, 11.89; N, 18.79.

#### 2-Bromo-N′-[quinazolin-4(3H)-ylidene]benzohydrazide (**3.26**)

Yield: Method A, 100%; M.p. 218–220°C (dioxane); ^1^H-NMR (400 MHz) δ: 10.17 (m, 2H, NH), 8.09 (d, 1H, H-6′), 8.08/7.92 (s, 1H, H-2), 7.69 (d, 1H, H-5), 7.61 (m, 2H, H-4′, H-6), 7.48 (t, 1H, H-3′), 7.40 (m, 2H, H-5′, H-8), 7.34/7.16 (t, 1H, H-7); LC-MS, *m/z* = 342 [M]; Anal. calcd. for C_15_H_11_BrN_4_O: C, 52.50; H, 3.23; Br, 23.28; N, 16.33; Found: C, 52.49; H, 3.21; Br, 23.26; N, 16.34.

#### 4-Bromo-N′-[quinazolin-4(3H)-ylidene]benzohydrazide (**3.27**)

Yield: Method B, 84%; M.p. 256–258°C (dioxane); ^1^H-NMR (400 MHz) δ: 10.7 (s, 2H, NH), 8.25 (d, 1H, *J*=8.0, H-5), 8.14 (s, 1H, H-2), 7.86 (d, 2H, *J*=7.8, H-2′,H-6′), 7.62 (d, 2H, *J*=7.8, H-3′,H-5′), 7.60 (t, 1H, *J*=7.9, H-7), 7.45 (d, 1H, *J*=8.0, H-8), 7.40 (t, 1H, *J*=7.9, H-6); LC-MS, *m/z* = 343 [M], 345 [M+2]; Anal. calcd. for C_15_H_11_BrN_4_O: C, 52.50; H, 3.23; Br, 23.28; N, 16.33; Found: C, 52.53; H, 3.25; Br, 23.31; N, 16.36.

#### 2-Nitro-N′-[quinazolin-4(3H)-ylidene]benzohydrazide (**3.28**)

Yield: Method B, 74%; M.p. 256–258°C (dioxane-H_2_O); ^1^H-NMR (400 MHz) δ: 11.48 (s, 1H, NH), 9.95 (s, 1H, NH), 8.05-7.0 (m, 9H, H_arom_); LC-MS, *m/z* = 310 [M+1]; Anal. calcd. for C_15_H_11_N_5_O_3_: C, 58.25; H, 3.58; N, 22.64; Found: C, 58.26; H, 3.59; N, 22.66.

#### 3-Nitro-N′-[quinazolin-4(3H)-ylidene]benzohydrazide (**3.29**)

Yield: Method B, 99%; M.p. >300°C (DMF); ^1^H-NMR (400 MHz) δ: 10.26 (m, 2H, NH), 8.75-7.4 (m, 9H, H_arom_); LC-MS, *m/z* = 310 [M+1]; Anal. calcd. for C_15_H_11_N_5_O_3_: C, 58.25; H, 3.58; N, 22.64; Found: C, 58.28; H, 3.61; N, 22.69.

#### 4-Nitro-N′-[quinazolin-4(3H)-ylidene]benzohydrazide (**3.30**)

Yield: Method B, 90%; M.p. >300°C (DMF); ^1^H-NMR (400 MHz) δ: 10.22 (m, 2H, NH), 8.4-7.1 (m, 9H, H_arom_); LC-MS, *m/z* = 310 [M+1]; Anal. calcd. for C_15_H_11_N_5_O_3_: C, 58.25; H, 3.58; N, 22.64; Found: C, 58.27; H, 3.59; N, 22.68.

#### N′-[Quinazolin-4(3H)-ylidene]-2-furohydrazide (**3.31**)

Yield: Method B, 78%; M.p. 226–228°C (dioxane–H_2_O); ^1^H-NMR (400 MHz) δ: 11.83 (s, 1H, NH), 10.79 (s, 1H, NH), 9.64 (s, 1H, NH), 8.61-6.53 (m, 8H, H_arom_, H-3′,. H-4′,. H-5′); LC-MS, *m/z* = 255 [M+1]; Anal. calcd. for C_13_H_10_N_4_O_2_: C, 61.41; H, 3.96; N, 22.04; Found: C, 61.40; H, 3.96; N, 22.06.

#### N′-[Quinazolin-4(3H)-ylidene]-3-furohydrazide (**3.32**)

Yield: Method B, 75%; M.p. 214–218°C (dioxane–H_2_O); ^1^H-NMR (400 MHz) δ: 11.74 (s, 1H, NH), 10.41/10.26 (s, 1H, NH), 8.74-6.62 (m, 8H, H_arom_, H-2′,. H-4′,. H-5′); LC-MS, *m/z* = 255 [M+1]; Anal. calcd. for C_13_H_10_N_4_O_2_: C, 61.41; H, 3.96; N, 22.04; Found: C, 61.42; H, 3.98; N, 22.07.

#### N′-[Quinazolin-4(3H)-ylidene]thiophene-2-carbohydrazide (**3.33**)

Yield: Method B, 53%; M.p. 222–226°C (dioxane–H_2_O); ^1^H-NMR (400 MHz) δ: 11.76 (s, 1H, NH), 10.67/9.94 (s, 1H, NH), 8.46-7.07 (m, 8H, H_arom_, H-3′,. H-4′,. H-5′); LC-MS, *m/z* = 271 [M+1]; Anal. calcd. for C_13_H_10_N_4_OS: C, 57.76; H, 3.73; N, 20.73; S, 11.86; Found: C, 57.74; H, 3.76; N, 20.76,; S, 11.88.

#### N′-[Quinazolin-4(3H)-ylidene]thiophene-3-carbohydrazide (**3.34**)

Yield: Method B, 68%; M.p. 218–220°C (dioxane-H_2_O); ^1^H-NMR (400 MHz) δ: 11.76 (s, 1H, NH), 10.54/10.30 (s, 1H, NH), 8.77-6.63 (m, 8H, H_arom_, H-2′,. H-4′, H-5′); LC-MS, *m/z* = 271 [M+1]; Anal. calcd. for C_13_H_10_N_4_OS: C, 57.76; H, 3.73; N, 20.73; S, 11.86; Found: C, 57.78; H, 3.76; N, 20.76,; S, 11.89.

#### N′-[Quinazolin-4(3H)-ylidene]-1H-pyrrole-2-carbohydrazide (**3.35**)

Yield: Method B, 73%; M.p. 170–172°C (dioxane-H_2_O); LC-MS, *m/z* = 254 [M+1]; Anal. calcd. for C_13_H_11_N_5_O: C, 61.65; H, 4.38; N, 27.65; Found: C, 61.68; H, 4.39; N, 27.67.

#### Ethyl 2,4-dimethyl-5-{[2-(quinazolin-4(3H)-ylidene)hydrazinyl]carbonyl}-1H-pyrrole-3-carboxylate (**3.36**)

Yield: Method B, 82%; M.p. 235–240°C (dioxane-H_2_O); ^1^H-NMR (400 MHz) δ: 12.22 (s, 1H, N_pir._), 10.24 (s, 2H, NH), 8.35 (m, 5H, H_arom_), 4.20 (q, 2H, *J* = 7.1, -CH_2_), 2.56 (s, 3H, 2-CH_3_), 2.44 (s, 3H, 4-CH_3_), 1.42 (t, 3H, *J* = 7.1, -CH_3_); LC-MS, *m/z* = 354 [M+1]; Anal. calcd. for C_18_H_19_N_5_O_3_: C, 61.18; H, 5.42; N, 19.82; Found: C, 61.18; H, 5.42; N, 19.82.

#### N′-[Quinazolin-4(3H)-ylidene]-1-benzofuran-2-carbohydrazide (**3.37**)

Yield: Method B, 63%; M.p. 286–290°C (dioxane-H_2_O); ^1^H-NMR (400 MHz) δ: 11.96 (s, 1H, NH), 11.03 (s, 1H, NH), 8.12 (s, 1H, H-3′), 8.00 (d, 1H, *J* = 7.9, H-5), 7.80 (d, 1H, *J* = 7.8, H-8), 7.75 (d, 1H, *J* = 8.4, H-7′.), 7.64 (s, 1H, H-2), 7.57 (t, 1H, *J* = 7.8, H-6), 7.50 (t, 1H, *J* = 7.8, H-7), 7.37 (m, 2H, H-5′, H-6′), 7.25 (d, 1H, *J* = 8.4, H-4′.); LC-MS, *m/z* = 305 [M+1]; Anal. calcd. for C_17_H_12_N_4_O_2_: C, 67.10; H, 3.97; N, 18.41; Found: C, 67.12; H, 3.99; N, 18.42.

#### N′-[Quinazolin-4(3H)-ylidene]-1-benzothiophene-2-carbohydrazide (**3.38**)

Yield: Method B, 84%; M.p. 136–138°C (dioxane-H_2_O); ^1^H-NMR (400 MHz) δ: 11.89 (s, 1H, NH), 10.91/10.20 (s, 1H, NH), 8.64-7.35 (m, 10H, H_arom_).; LC-MS, *m/z* = 321 [M+1]; Anal. calcd. for C_17_H_12_N_4_OS: C, 63.73; H, 3.78; N, 17.49; S, 10.01; Found: C, 63.72; H, 3.76; N, 17.46; S, 9.99.

#### N′-[Quinazolin-4(3H)-ylidene]-1H-indole-2-carbohydrazide (**3.39**)

Yield: Method B, 57%; M.p. 182–186°C (dioxane-H_2_O); ^1^H-NMR (400 MHz) δ: 11.83 (s, 1H, NH), 11.76 (s, 1H, NH), 10.77 (s, 1H, -NH), 7.72-6.99 (m, 10H, H_arom_); LC-MS, *m/z* = 304 [M+1]; Anal. calcd. for C_17_H_13_N_5_O: C, 67.32; H, 4.32; N, 23.09; Found: C, 67.34; H, 4.36; N, 23.12.

#### N′-[Quinazolin-4(3H)-ylidene]nicotinohydrazide (**3.40**)

Yield: Method A, 80; M.p. 249–251°C (DMF); ^1^H-NMR (400 MHz) δ: 10.71 (m, 2H, NH), 9.06 (s, 1H, H-2′), 8.70 (d, 1H, *J* = 4.6, H-4′), 8.25-8.19 (m, 2H, H-5, H-5′), 8.11 (s, 1H, H-2), 7.60 (t, 1H, *J* = 7.8, H-7), 7.49 (d, 1H, *J* = 8.0, H-8), 7.46 (d, 1H, *J* = 4.6, H-6′), 7.40 (t, 1H, *J* = 7.8, H-6); LC-MS, *m/z* = 266 [M+1]; Anal. calcd. for C_14_H_11_N_5_O: C, 72.56; H, 4.18; N, 26.40; Found: C, 72.54; H, 4.16; N, 26.38.

#### N′-[Quinazolin-4(3H)-ylidene]isonicotinohydrazide (**3.41**)

Yield: Method B, 94%; M.p. 254–256°C (DMF); ^1^H-NMR (400 MHz) δ: 10.79 (m, 2H, NH), 8.72, 7.78 (d, 2H, *J* = 5.0, H-2′, H-3′, H-5′, H-6′), 8.18 (d, 1H, *J* = 8.0, H-5), 8.07 (s, 1H, H-2), 7.58 (t, 1H, *J* = 7.8, H-7), 7.4-7.3 (m, 2H, H-6, H-8); LC-MS, *m/z* = 266 [M+1]; Anal. calcd. for C_14_H_11_N_5_O: C, 72.56; H, 4.18; N, 26.40; Found: C, 72.57; H, 4.19; N, 26.42.

### General procedure for N-(3H-Quinazolin-4-ylidene)hydrazone benzaldehydes (4.1–4.6)

To 1.60 g (10 mM) of 4-hydrazinoquinazoline (**2.1**) in 10–15 ml of 2-propanol or dioxane, the corresponding benzaldehyde (0.01 M) was added. The mixture was refluxed for 1–1.5 h, and cooled. The formed precipitate was filtered and dried.

#### N-[Quinazolin-4(3H)-ylidene]hydrazone benzaldehyde (**4.1**)

Yield: 89%; M.p. 176–178°C (propanol); ^1^H-NMR (400 MHz) δ: 11.72 (s, 1H, NH), 8.53 (s,1H, -CH=N), 8.22 (d, 1H, H-5), 7.92-7.87 (m., 2H, H-2′, H-6′), 7.86 (s, 1H, H-2), 7.65 (t, 1H, H-7), 7.60-7.55 (m, 3H, H-3′, H-4′, H-5′), 7.54 (d, 1H, H-8), 7.45 (t, 1H, H-6), LC-MS, *m/z* = 249 [M+1]; Anal. calcd. for C_15_H_12_N_4_: C, 72.56; H, 4.87; N, 22.57; Found: C, 72.54; H, 4.86; N, 22.55.

#### N-[Quinazolin-4(3H)-ylidene]hydrazone 2-hydroxybenzaldehyde (**4.2**)

Yield: 95%; M.p. 182–184°C (2-propanol-H_2_O); ^1^H-NMR (400 MHz) δ: 11.68 (s, 1H, NH), 8.76 (s, 1H, -CH=N), 8.24 (d, 1H, H-5), 7.88 (m, 2H, H-2, H-6′), 7.70 (t, 1H, H-7), 7.60-7.40 (m, 2H, H-8, H-6), 7.32 (t, 1H, H-4′), 6.94 (d, 2H, H-3′, H-5′); LC-MS, *m/z* = 265 [M+1]; Anal. calcd. for C_15_H_12_N_4_O: C, 68.17; H, 4.58; N, 21.20; Found: C, 68.18; H, 4.59; N, 21.19.

#### N-[Quinazolin-4(3H)-ylidene]hydrazone 4-hydroxybenzaldehyde (**4.3**)

Yield: 83%; M.p. 258–260°C (dioxane); ^1^H-NMR (400 MHz) δ: 11.53 (c.,1H, NH), 9.93 (s, 1H, -OH), 8.42 (s,1H, -CH=N), 8.14 (d, 1H, H-5), 7.87 (d., 2H, H-4′, H-6′), 7.81 (s, 1H, H-2), 7.63 (t, 1H, H-7), 7.48 (d, 1H, H-8), 7.39 (t,1H, H-6), 6.87 (d. 2H, H-3′, H-5′); LC-MS, *m/z* = 265 [M+1]; Anal. calcd. for C_15_H_12_N_4_O: C, 68.17; H, 4.58; N, 21.20; Found: C, 68.15; H, 4.56; N, 21.17.

#### N-[Quinazolin-4(3H)-ylidene]hydrazone 2-methoxybenzaldehyde (**4.4**)

Yield: 90%; M.p. 184–186°C (2-propanol–H_2_O); ^1^H-NMR (400 MHz) δ: 11.27 (c.,1H, NH), 8.79 (s,1H, -CH=N), 8.26 (d., 1H, H-6′), 8.24 (d, 1H, H-5), 7.74 (s, 1H, H-2), 7.53 (t, 1H, H-7), 7.45 (d, 1H, H-8), 7.34 (t,1H, H-6), ; 7.43 (d., 1H, H-3′.), 6.97 (m, 2H, H-4′, H-5′), 3.93 (s., 3H, 2-OCH_3_); LC-MS, *m/z* = 279 [M+1]; Anal. calcd. for C_16_H_14_N_4_O: C, 69.05; H, 5.07; N, 20.13; Found: C, 69.07; H, 5.09; N, 20.16.

#### N-[Quinazolin-4(3H)-ylidene]hydrazone 4-chlorobenzaldehyde (**4.5**)

Yield: 75%; M.p. 166–168°C (propanol); ^1^H-NMR (400 MHz) δ: 11.73 (s.,1H, NH), 8.54 (s,1H, -CH=N), 8.22 (d, 1H, H-5), 8.00 (d., 2H, H-2′, H-6′), 7.95 (s, 1H, H-2), 7.69 (t, 1H, H-7), 7.54 (d, 1H, H-8), 7.53 (d. 2H, H-3′, H-5′), 7.46 (t,1H, H-6); LC-MS, *m/z* = 283 [M+1], 285 [M+3]; Anal. calcd. for C_15_H_11_ClN_4_: C, 63.72; H, 3.92; Cl, 12.54; N, 19.82; Found: C, 63.73; H, 3.90; Cl, 12.56; N, 19.84.

#### N-[Quinazolin-4(3H)-ylidene]hydrazone 4-bromobenzaldehyde (**4.6**)

Yield: 90%; M.p. 248–250°C (propanol); ^1^H-NMR (400 MHz) δ: 11.75 (s.,1H, NH), 8.52 (s,1H, -CH=N), 8.19 (d, 1H, H-5), 7.94 (s, 1H, H-2), 7.88 (d., 2H, H-2′, H-6′), 7.69 (t, 1H, H-7), 7.66 (д., 2H, H-3′, H-5′), 7.52 (d, 1H, H-8), 7.45 (t,1H, H-6), LC-MS, *m/z* = 327 [M], 329 [M+2]; Anal. calcd. for C_15_H_11_BrN_4_: C, 55.06; H, 3.39; Br, 24.42; N, 17.12; Found: C, 55.08; H, 3.41; Br, 24.45; N, 17.14.

### General procedure for 2-R-[1,2,4]triazolo[1,5-c]quinazolines (5.1–5.41)

*Method A.* To 10 mM of the alkyl(aralkyl-, aryl-, hetaryl-)carboxylic acid [3*H*-quinazolin-4-ylidene]hydrazides (**3.1–3.41**), 10 ml of glacial acetic acid was added and refluxed for 3–6 h. The solvent was distilled off and water or a water-alcohol mixture was added to the residue. The formed precipitate was filtered off and dried.

*Method B.* (**5.12, 5.17, 5.23, 5.25, 5.27)** To a solution of 2 mM of N-(R-benzyliden)-N′-(3*H*-quinazolin-4-ylidene)hydrazine (**4.1–4.6**) in 20 ml of glacial acetic acid, 0.5 g (6 mM) of sodium acetate was added with stirring at room temperature. Then a solution of 0.32 g of bromine (2 mM) in 10 ml of glacial acetic acid was added dropwise to a mixture. The mixture was stirred for 1 hour, then was poured into crushed ice (100.0 g). The formed precipitate was filtered off and dried.

*Method C*. (**5.7, 5.12, 5.17, 5.18, 5.23, 5.23, 5.27**) 1.18 g (10 mM) of *o*-aminobenzonitrile (**1.1a**) in 5 ml of triethyl orthoformate was refluxed for 3 h, and after this the mixture was distilled off. To the resulting mixture, 10 mM of the corresponding carboxylic acid hydrazides and 5 ml of glacial acetic acid were added and refluxed for 4 h. The solvent was distilled off and methanol was added. The mixture was stirred for 30 min. The formed precipitates were filtered off and dried.

#### 2-Methyl-[1,2,4]triazolo[1,5-c]quinazoline (**5.1**)

Yield: Method A, 92%; M.p. 128–130°C (2-propanol); ^1^H-NMR (400 MHz) δ: 9.25 (s, 1H, H-5), 8.42 (d, 1H, *J*=8.0, H-10), 8.01 (d, 1H, *J*=8.0, H-7), 7.84 (t, 1H, *J*=7.8, H-8), 7.75 (t, 1H, *J*=7.9, H-9), 2.65 (s, 3H, C*H*_3_); EI-MS, *m/z* (I_rel_, %) = 185 (9.0), 184 (M^+•^, 100.0), 174 (13.6), 143 (8.7), 129 (8.8), 116 (11.5), 115 (14.3), 114 (6.2), 104 (8.2), 102 (13.2), 88 (12.4), 85 (7.3), 83 (10.4), 78 (6.4), 77 (5.3), 76 (7.8), 75 (6.4), 68 (9.6), 62 (11.6), 57 (7.8), 56 (37.5), 51 (6.0); LC-MS, *m/z* = 185 [M+1]; Anal. calcd. for C_10_H_8_N_4_: C, 65.21; H, 4.38; N, 30.42; Found: C, 65.19; H, 4.37; N, 30.40.

#### 2-Ethyl-[1,2,4]triazolo[1,5-c]quinazoline (**5.2**)

Yield: Method A, 93%; M.p. 84–86°C (2-propanol); ^1^H-NMR (400 MHz) δ: 9.26 (s, 1H, H-5), 8.42 (d, 1H, *J*=8.0, H-10), 7.99 (d, 1H, *J*=7.9, H-7), 7.82 (t, 1H, *J*=7.9, H-8), 7.71 (т, 1H, *J*=8.0, H-9), 2.08 (к, 2H, *J*=7.2, C*H*_2_CH_3_), 1.40 (т, 3H, *J*=7.2, C*H*_2_CH_3_); ^13^C NMR (125 MHz) δ: 168.86 (C-2), 150.60 (C-6a), 142.74 (C-10b), 139.20 (C-5), 132.50 (C-8), 129.37 (C-7), 128.88 (C-9), 123.62 (C-10), 117.87 (C-10a), 22.11 (-*C*H_2_-CH_3_), 12.60 (-CH_2_-*C*H_3_); EI-MS, *m/z* (I_rel_, %) = 199 (5), 198 (M^+•^, 40.0), 197 (52), 129 (30), 128 (6), 117 (6), 116 (10), 115 (9), 114 (10), 111 (9), 103 (8), 102 (32), 101 (6), 90 (10), 89 (12), 88 (16), 87 (6), 85 (8), 83 (8), 76 (10), 75 (12), 71 (100), 69 (14), 63 (8), 62 (16), 57 (6), 56 (15), 55 (12), 51 (8), 44 (6), 43 (43), 41 (19), 40 (6), 39 (12), 32 (9), 28 (32); LC-MS, *m/z* = 199 [M+1]; Anal. calcd. for C_11_H_10_N_4_: C, 66.65; H, 5.08; N, 28.26; Found: C, 66.68; H, 5.59; N, 28.29.

#### 2-Propyl-[1,2,4]triazolo[1,5-c]quinazoline (5.3)

Yield: Method A, 63%; M.p. 80–82°C (2-propanol); ^1^H-NMR (400 MHz) δ: 9.25 (s, 1H, H-5), 8.46 (d, 1H, *J* = 8.0, H-10), 8.00 (d, 1H, *J* = 7.9, H-7), 7.83 (t, 1H, *J* = 8.0, H-8), 7.71 (t, 1H, *J* = 7.8, H-9), 2.90 (t, 2H, *J* = 7.1, C*H*_2_CH_2_CH_3_), 1.88 (m, 2H, CH_2_C*H*_2_CH_3_), 1.02 (t, 3H, *J* = 7.1, CH_3_); LC-MS, *m/z* = 213 [M+1]; Anal. calcd. for C_12_H_12_N_4_: C, 67.90; H, 5.70; N, 26.40; Found: C, 67.88; H, 5.69; N, 26.38.

#### 2-(Chloromethyl)-[1,2,4]triazolo[1,5-c]quinazoline (**5.4**)

Yield: Method A, 66%; M.p. 153–155°C (2-propanol); ^1^H-NMR (400 MHz) δ: 9.64 (s, 1H, H-5), 8.46 (d, 1H, *J* = 8.1, H-10), 8.05 (d, 1H, *J* = 8.0, H-7), 7.96 (t, 1H, *J* = 8.0, H-8), 7.83 (t, 1H, *J* = 7.9, H-9), 5.03 (s, 2H, CH_2_); ^13^C-NMR (125 MHz) δ: 163.40 (C-2), 151.14 (C-6a), 142.82 (C-10b), 139.37 (C-5), 132.98 (C-8), 129.73 (C-7), 129.04 (C-9), 123.76 (C-10), 117.88 (C-10a), 38.24 (-*C*H_2_-Cl); LC-MS, *m/z* = 219 [M+1], 221 [M+3]; Anal. calcd. for C_10_H_7_ClN_4_: C, 54.93; H, 3.23; Cl, 16.21; N, 25.62; Found: C, 54.96; H, 3.24; Cl, 16.23; N, 25.63.

#### 2-[(2,4,5-Trichlorophenoxy)methyl][1,2,4]triazolo[1,5-c]quinazoline (**5.5**)

Yield: Method A, 88%; M.p. 204–206°C (DMF–H_2_O); ^1^H-NMR (400 MHz) δ: 9.68 (s, 1H, H-5), 8.48 (d, 1H, *J* = 8.0, H-10), 8.07 (d, 1H, *J* = 8.0, H-7), 7.95 (t, 1H, *J* = 7.9, H-8), 7.86 (t, 1H, *J* = 8.0, H-9), 7.84 (s, 1H, H-6′), 7.74 (s, 1H, H-3′), 5.67 (s, 2H, CH_2_); LC-MS, *m/z* = 379 [M], 381 [M+1], 383 [M+4], 385 [M+5]; Anal. calcd. for C_16_H_9_Cl_3_N_4_O: C, 50.62; H, 2.39; Cl, 28.02; N, 14.76; Found: C, 50.60; H, 2.38; Cl, 28.03; N, 14.78.

#### ([1,2,4]Triazolo[1,5-c]quinazolin-2-yl)acetonitrile (**5.6**)

Yield: Method A, 67%; M.p. 184–186°C (DMF-H_2_O); ^1^H-NMR (400 MHz) δ: 9.85 (s, 1H, H-5), 8.62 (d, 1H, *J* = 8.1, H-10), 8.25 (d, 1H, *J* = 8.0, H-7), 7.96 (t, 1H, *J* = 8.0, H-8), 7.86 (t, 1H, *J* = 7.9, H-9), 4.63 (s, 2H, CH_2_); LC-MS, *m/z* = 210 [M+1]; Anal. calcd. for C_10_H_7_N_5_: C, 63.15; H, 3.37; N, 33.48; Found: C, 63.16; H, 3.33; N, 33.49.

#### 2-Benzyl[1,2,4]triazolo[1,5-c]quinazoline (**5.7**)

Yield: Method A, 92%; Method C, 42.6%; M.p. 166–168°C (ethanol); ^1^H-NMR (400 MHz) δ: 9.31 (s, 1H, H-5), 8.44 (d, 1H, *J*=8.0, H-10), 7.98 (d, 1H, *J*=8.1, H-7), 7.82 (t, 1H, *J*=7.9, H-8), 7.71 (t, 1H, *J*=7.8, H-9), 7.37 (d, 2H, *J*=7.8, H-2′, H-6′), 7.28 (t, 2H, *J*=7.8, H-3′, H-5′), 7.16 (t, 1H, *J*=7.9, H-4′), 4.25 (s, 2H, C*H*_2_); EI-MS, *m/z* (I_rel_, %) = 261 (16.6), 260 (M^+•^, 89.6), 259 (100), 258 (6.0), 132 (14.5), 131 (20.0), 130 (11.0), 129 (15.8), 117 (5.5), 116 (17.6), 104 (15.4), 103 (22.0), 102 (19.9), 91 (28.0), 90 (8.9), 89 (15.4), 88 (7.5), 77 (16.1), 76 (7.8), 75 (6.1), 65 (19.1), 63 (9.2), 62 (7.2), 51 (10.6); LC-MS, *m/z* = 261 [M+1]; Anal. calcd. for C_16_H_12_N_4_: C, 73.83; H, 4.65; N, 21.52; Found: C, 73.85; H, 4.67; N, 21.53.

#### Diphenyl([1,2,4]triazolo[1,5-c]quinazolin-2-yl)methanol (**5.8**)

Yield: Method A, 65%; M.p. 143–145°C (DMF–H_2_O); ^1^H-NMR (400 MHz) δ: 9.41 (s, 1H, H-5), 8.48 (d, 1H, *J* = 8.1, H-10), 8.03 (d, 1H, *J* = 8.0, H-7), 7.85 (t, 1H, *J* = 7.8, H-8), 7.72 (t, 1H, *J* = 7.8, H-9), 7.45 (m, 4H, Ph), 7.23 (m, 6H, Ph), 6.25 (c, 1H, OH); LC-MS, *m/z* = 353 [M+1]; Anal. calcd. for C_22_H_16_N_4_O: C, 74.98; H, 4.58; N, 15.90; Found: C, 74.95; H, 4.55; N, 15.88.

#### 2-(Phenethyl)[1,2,4]triazolo[1,5-c]quinazoline (**5.9**)

Yield: Method A, 78%; Method B, 76.3%; M.p. 112–114°C (ethanol–H_2_O); ^1^H-NMR (400 MHz) δ: 9.52 (s, 1H, H-5), 8.42 (d, 1H, *J*=8.0, H-10), 8.03 (d, 1H, *J*=8.0, H-7), 7.91 (t, 1H, *J*=7.8, H-8), 7.80 (t, 1H, *J*=7.8, H-9), 7.28 (m, 4H, H-2′,H-3′, H-5′,H-6′), 7.17 (m, 1H, H-4′), 3.20-3.18 (m., 4H, (C*H*_2_C*H*_2_Ph); EI-MS, *m/z* (I_rel_, %) = 275 (11.1), 274 (M^+•^, 62.3), 273 (69.2), 197 (39.4), 129 (12.3), 128 (5.0), 104 (8.9), 103 (8.1), 102 (14.2), 92 (6.9), 91 (100), 77 (8.9), 65 (18.6), 51 (5.1); LC-MS, *m/z* = 275 [M+1], 276 [M+2]; Anal. calcd. for C_17_H_12_N_4_: C, 74.43; H, 5.14; N, 20.42; Found: C, 74.40; H, 5.13; N, 20.40.

#### 2-[(E)-2-Phenylvinyl][1,2,4]triazolo[1,5-c]quinazoline (**5.10**)

Yield: Method A, 36%; Method B, 91.6%, 66.1%; M.p. 216–218°C (dioxane-H_2_O); ^1^H-NMR (400 MHz) δ: 9.58 (s, 1H, H-5), 8.48 (d, 1H, *J* = 7.8, H-10), 8.07 (d, 1H, *J* = 7.8, H-7), 7.95 (t, 1H, *J* = 7.8, H-8), 7.88 (d, 1H, *J* = 16.4, CH=C*H*-Ph), 7.84 (t, 1H, *J* = 7.8, H-9), 7.81 (d, 2H, *J* = 7.8, H-2′,6′), 7.48–7.35 (m, 4H, H-3′,5′, C*H*=CH-Ph); LC-MS, *m/z* = 273 [M+1]; Anal. calcd. for C_17_H_12_N_4_: C, 74.98; H, 4.44; N, 20.58; Found: C, 74.97; H, 4.43; N, 20.59.

#### 2-(1H-Indol-3-ylmethyl)[1,2,4]triazolo[1,5-c]quinazoline (**5.11**)

Yield: Method A, 88%; M.p. 212–214°C (dioxane–H_2_O); ^1^H-NMR (400 MHz) δ: 10.68 (s, 1H, NH), 9.28 (s, 1H, H-5), 8.44 (d, 1H, *J* = 8.0, H-10), 7.98 (d, 1H, *J* = 8.1, H-7), 7.82 (t, 1H, *J* = 7.8, H-8), 7.72 (t, 1H, *J* = 7.8, H-9), 7.58 (d, 1H, *J* = 7.8, H-7′), 7.31 (d, 1H, *J* = 7.8, H-4′), 7.21 (s, 1H, H-2′), 7.03 (t, 1H, *J* = 7.8, H-5′), 6.92 (t, 1H, *J* = 7.8, H-6′), 4.47 (s, 2H, CH_2_); LC-MS, *m/z* = 300 [M+1]; Anal. calcd. for C_18_H_13_N_5_: C, 72.23; H, 4.38; N, 23.40; Found: C, 72.26; H, 4.41; N, 23.42.

#### 2-Phenyl[1,2,4]triazolo[1,5-c]quinazoline (**5.12**)

Yield: Method A, 89%; Method B, 42.9%; Method C, 68.9%; M.p. 202–204°C (dioxane– H_2_O); ^1^H-NMR (400 MHz) δ: 9.68 (s, 1H, H-5), 8.52 (d, 1H, *J*=8.0, H-10), 8.27 (d, 2H, H-2′, H-6′), 8.07 (d, 1H, *J*=8.0, H-7), 7.94 (t, 1H, *J*=7.8, H-8), 7.84 (t, 1H, *J*=7.8, H-9), 7.56 (m, 3H, H-3′, H-4′, H-5′); ^13^C NMR (125 MHz) δ: 164.06 (C-2), 151.27 (C-6a), 142.90 (C-10b), 140.01 (C-5), 139.50 (C-8), 132.81 (C-1 Ph.), 131.20 (C-4 Ph.), 130.27 (C-7), 129.57 (C-3 Ph., C-5 Ph.), 129.03 (C-9), 127.53 (C-2 Ph., C-6 Ph.), 123.85 (C-10), 118.05 (C-10a); EI-MS, *m/z* (I_rel_, %) = 248 (1.2), 247 (14.8), 246 (M^+•^, 100), 245 (41.6), 129 (6.4), 118 (18.6), 116 (8.7), 115 (6.0), 114 (7.2), 109 (5.6), 102 (9.3), 91 (7.6), 90 (6.1), 89 (12.1), 88 (15.1), 77 (13.7), 76 (5.5), 63 (7.9), 62 (12.5), 51 (7.5); LC-MS, *m/z* = 247 [M+1]; Anal. calcd. for C_15_H_10_N_4_: C, 73.16; H, 4.09; N, 22.75; Found: C, 73.15; H, 4.07; N, 22.73.

#### 2-(3-Methylphenyl)[1,2,4]triazolo[1,5-c]quinazoline (**5.13**)

Yield: Method A, 92%; M.p. 224–226°C (dioxane-H_2_O); ^1^H-NMR (400 MHz) δ: 9.59 (s, 1H, H-5), 8.50 (d, 1H, *J*^3^= 7.9, *J*^4^= 1.6, H-10), 8.10-8.02 (m, 3H, H-7, H-2′, H-6′), 7.97-7.78 (m, 2H, H-8, H-9), 7.48-7.32 (m, 2H, H-4′, H-5′), 2.42 (s, 3H, C*H*_3_); LC-MS, *m/z* = 261 [M+1], 262 [M+2]; Anal. calcd. for C_16_H_12_N_4_: C, 73.83; H, 4.65; N, 21.52; Found: C, 73.81; H, 4.63; N, 21.52.

#### 2-(4-Methylphenyl)[1,2,4]triazolo[1,5-c]quinazoline (**5.14**)

Yield: Method A, 93%; M.p. 276–278°C (dioxane-H_2_O); ^1^H-NMR (400 MHz) δ: 9.36 (s, 1H, H-5), 8.56 (d, 1H, *J*=8.1, H-10), 8.19 (d, 2H, *J*=8.0, H-2′, H-6′), 8.05 (d, 1H, *J*=8.1, H-7), 7.88 (t, 1H, *J*=8.0, H-8), 7.76 (t, 1H, *J*=7.9, H-9), 7.28 (d, 2H, *J*=8.0, H-3′, H-5′), 2.45 (s, 3H, C*H*_3_); EI-MS, *m/z* (I_rel_, %) = 261 (15.6), 260 (M^+•^, 100), 259 (28.2), 132 (11.1), 131 (12.8), 129 (7.4), 116 (15.3), 115 (5.7), 114 (5.5), 104 (5.4), 103 (7.4), 102 (12.6), 91 (13.6), 90 (8.9), 89 (12.1), 88 (15.0), 77 (8.1), 65 (8.7), 63 (6.6), 62 (10.7), 51 (5.4); LC-MS, *m/z* = 261 [M+1]; Anal. calcd. for C_16_H_12_N_4_: C, 73.83; H, 4.65; N, 21.52; Found: C, 73.86; H, 4.68; N, 21.55.

#### 2-[4-(Trifluoromethyl)phenyl][1,2,4]triazolo[1,5-c]quinazoline (**5.15**)

Yield: Method A, 86%; M.p. 220–224°C (dioxane-H_2_O); ^1^H-NMR (400 MHz) δ: 9.63 (s, 1H, H-5), 8.49-8.41 (m, 3H, H-10, H-2′, H-6′), 8.05 (d, 1H, *J* = 8.1, H-7), 7.96-7.76 (m, 4H, H-8, H-9, H-3′, H-5′); EI-MS, *m/z* (I_rel_, %) = 315 (15.9), 314 (M^+•^, 100), 313 (41.7), 305 (5.4), 186 (10.3), 173 (5.0), 145 (5.5), 143 (6.7), 129 (7.8), 120 (7.4), 118 (4.6), 116 (8.2), 115 (11.1), 114 (9.4), 102 (12.9), 89 (6.6), 88 (19.2), 87 (6.2), 85 (8.7), 83 (11.3), 76 (5.0), 75 (6.4), 62 (12.3), 51 (4.0); LC-MS, *m/z* = 315 [M+1]; Anal. calcd. for C_16_H_9_F_3_N_4_: C, 61.15; H, 2.89; F, 18.14; N, 17.83; Found: C, 61.18; H, 2.92; F, 18.16; N, 17.86.

#### 2-(2-Hydroxyphenyl)[1,2,4]triazolo[1,5-c]quinazoline (**5.16**)

Yield: Method A, 81%; M.p. 210–−212°C (2-propanol–H_2_O); ^1^H-NMR (400 MHz) δ: 10.97 (s, 1H, OH), 9.74 (s, 1H, H-5), 8.62 (d, 1H, *J* = 8.1, H-10), 8.19 (d, 1H, *J* = 7.8, H-6′), 8.13 (d, 1H, *J* = 8.0, H-7), 8.01 (t, 1H, *J* = 8.0, H-8), 7.89 (t, 1H, *J* = 7.8, H-9), 7.45 (t, 1H, *J* = 8.0, H-4′), 7.08 (m, 2H, H-3′,5′); LC-MS, *m/z* = 263 [M+1]; Anal. calcd. for C_15_H_10_N_4_O: C, 68.69; H, 3.84; N, 21.36; Found: C, 68.71; H, 3.86; N, 21.39.

#### 2-(4-Hydroxyphenyl)[1,2,4]triazolo[1,5-c]quinazoline (**5.17**)

Yield: Method A, 95%; Method B, 25.3%; Method C, 73.2%; M.p. >300°C (DMF–H_2_O); ^1^H-NMR (400 MHz) δ: 9.60 (s, 1H, H-5), 8.51 (d, 1H, *J*=8.0, H-10), 8.11 (d, 2H, *J*=7.8, H-2′, H-6′), 8.06 (d, 1H, *J*=8.0, H-7), 7.95 (t, 1H, *J*=8.0, H-8), 7.87 (t, 1H, *J*=8.0, H-9), 6.94 (d, 2H, *J*=7.8, H-3′, H-5′); ^13^C NMR (125 MHz) δ: 164.40 (C-1 Ph.), 160.25 (C-2), 151.06 (C-6a), 142.88 (C-10b), 139.33 (C-5), 132.59 (C-8), 129.39 (C-7), 129.27 (C-3 Ph., C-5 Ph.), 128.94 (C-9), 123.77 (C-10), 121.08 (C-4 Ph.), 117.94 (C-10a), 116.28 (C-2 Ph., C-6 Ph.); LC-MS, *m/z* = 263 [M+1]; Anal. calcd. for C_15_H_10_N_4_O: C, 68.69; H, 3.84; N, 21.36; Found: C, 68.67; H, 3.83; N, 21.34.

#### 2-(2-Methoxyphenyl)[1,2,4]triazolo[1,5-c]quinazoline (**5.18**)

Yield: Method A, 94%; Method C, 53.2%; M.p. 142–144°C (dioxane–H_2_O); EI-MS, *m/z* (I_rel_, %) = 277 (3.6), 276 (M^+•^, 13.8), 275 (17.0), 262 (16.1), 261 (9.2), 257 (5.6), 248 (20.7), 247 (100), 246 (16.2), 245 (34.5), 232 (19.1), 231 (11.8), 221 (5.9), 220 (5.0), 159 (6.5), 147 (9.1), 146 (6.0), 145 (57.8), 144 (9.3), 130 (8.3), 129 (27.1), 119 (8.8), 118 (41.6), 117 (5.2), 116 (8.0), 115 (8.8), 114 (6.3), 105 (6.3), 104 (11.2), 103 (12.0), 102 (27.8), 91 (15.0), 90 (14.9), 89 (14.8), 88 (23.1), 81 (6.2), 78 (7.8), 77 (23.8), 76 (11.8), 75 (10.7), 65 (6.0), 64 (9.1), 63 (12.7), 62 (13.4), 51 (8.5); LC-MS, *m/z* = 277 [M+1]; Anal. calcd. for C_16_H_12_N_4_O: C, 69.55; H, 4.38; N, 20.28; Found: C, 69.53; H, 4.36; N, 20.25.

#### 2-(4-Methoxyphenyl)[1,2,4]triazolo[1,5-c]quinazoline (**5.19**)

Yield: Method A, 83%; M.p. 194–196°C (dioxane–H_2_O); ^1^H-NMR (400 MHz) δ: 9.56 (s, 1H, H-5), 8.48 (d, 1H, *J*=8.0, H-10), 8.20 (d, 2H, *J*=8.3, H-2′,H-6′), 8.05 (d, 1H, *J*=8.1, H-7), 7.92 (t, 1H, *J*=7.6, H-8), 7.81 (t, 1H, *J*=7.6, H-9), 8.20 (d, 2H, *J*=8.3, H-3′, H-5′), 3.84 (s, 3H, OC*H*_3_); EI-MS, *m/z* (I_rel_, %) = 278 (1.7), 277 (17.4), 276 (M^+•^, 100), 275 (7.7), 261 (14.5), 233 (26.8), 133 (9.2), 129 (7.1), 105 (8.3), 102 (9.8), 90 (6.9), 88 (8.7), 77 (10.0), 76 (5.6), 63 (5.7), 62 (7.1), 51 (5.6); LC-MS, *m/z* = 277 [M+1]; Anal. calcd. for C_16_H_12_N_4_O: C, 69.55; H, 4.38; N, 20.28; Found: C, 69.58; H, 4.39; N, 20.31.

#### 2-(3,4,5-Trimethoxyphenyl)[1,2,4]triazolo[1,5-c]quinazoline (**5.20**)

Yield: Method A, 60%; M.p. 256–258°C (dioxane–H_2_O); ^1^H-NMR (400 MHz) δ: 9.35 (s, 1H, H-5), 8.60 (d, 1H, *J* = 8.0, H-10), 8.04 (d, 1H, *J* = 8.0, H-7), 7.86 (t, 1H, *J* = 7.9, H-8), 7.75 (t, 1H, *J* = 7.9, H-9), 7.54 (s, 2H, H-2′,6′), 3.97 (s, 6H, 3,5-(OCH_3_)_2_), 3.81 (s, 3H, 4-OCH_3_); LC-MS, *m/z* = 337 [M+1]; Anal. calcd. for C_18_H_16_N_4_O_3_: C, 64.28; H, 4.79; N, 16.66; Found: C, 64.29; H, 4.81; N, 16.68.

#### 2-(2-Fluorophenyl)[1,2,4]triazolo[1,5-c]quinazoline (**5.21**)

Yield: Method A, 73%; M.p. 184–186°C (2-propanol–H_2_O); ^1^H-NMR (400 MHz) δ: 9.70 (s, 1H, H-5), 8.52 (d, 1H, *J*=8.1, H-10), 8.30 (t, 1H, *J*=7.8, H-6′), 8.09 (d, 1H, *J*=8.1, H-7), 7.96 (t, 1H, *J*=7.8, H-8), 7.85 (t, 1H, *J*=7.8, H-9), 7.62 (m, 1H, H-4′), 7.44 (m, 2H, H-3′, H-5′); LC-MS, *m/z* = 265 [M+1]; Anal. calcd. for C_15_H_9_FN_4_: C, 68.18; H, 3.43; F, 7.19; N, 21.20; Found: C, 68.18; H, 3.40; F, 7.17; N, 21.18.

#### 2-(4-Fluorophenyl)[1,2,4]triazolo[1,5-c]quinazoline (**5.22**)

Yield: Method A, 79%; M.p. 268–269°C (dioxane–H_2_O); ^1^H-NMR (400 MHz) δ: 9.62 (s, 1H, H-5), 8.52 (d, 1H, *J*=7.6, H-10), 8.31 (m, 2H, H-2′, H-6′), 8.08 (d, 1H, *J*=7.6, H-7), 7.98-7.80 (m, 2H, H-8, H-9), 7.40 (m, 2H, H-3′, H-5′); EI-MS, *m/z* (I_rel_, %) = 266 (1.1), 265 (15.2), 264 (M^+•^, 100), 263 (42.3), 136 (18.9), 129 (7.3), 118 (6.0), 116 (7.1), 115 (9.5), 114 (8.2), 109 (8.9), 107 (6.7), 102 (8.3), 88 (17.6), 75 (6.8), 62 (12.6); LC-MS, *m/z* = 265 [M+1], 266 [M+2]; Anal. calcd. for C_15_H_9_FN_4_: C, 68.18; H, 3.43; F, 7.19; N, 21.20; Found: C, 68.15; H, 3.40; F, 7.15; N, 21.18.

#### 2-(2-Chlorophenyl)[1,2,4]triazolo[1,5-c]quinazoline (**5.23**)

Yield: Method A, 84%; Method B, 73.2%; Method C, 63.6%; M.p. 168–170°C (2-propanol– H_2_O); ^1^H-NMR (400 MHz) δ: 9.72 (s, 1H, H-5), 8.54 (d, 1H, *J*=8.0, H-10), 8.11 (m, 2H, H-7, H-6′), 7.99 (t, 1H,*J*=8.0, H-8), 7.69 (t, 1H, H-9), 7.73-7.54 (m, 3H, H-3′, H-4′, H-5′); LC-MS, *m/z* = 281 [M+1], 283 [M+3]; Anal. calcd. for C_15_H_9_ClN_4_: C, 64.18; H, 3.23; Cl, 12.63; N, 19.96; Found: C, 64.16; H, 3.21; Cl, 12.60; N, 19.98.

#### 2-(3-Chlorophenyl)[1,2,4]triazolo[1,5-c]quinazoline (**5.24**)

Yield: Method B, 90%; M.p. 224–226°C (dioxane–H_2_O); ^1^H-NMR (400 MHz) δ: 9.65 (s, 1H, H-5), 8.52 (d, 1H, *J* =8.0, H-10), 8.22 (m, 2H, H-2′, H-6′), 7.61 (m, 2H, H-4′, H-5′), 8.08 (d, 1H, *J*=8.0, H-7), 7.96 (t, 1H, *J*=7.8, H-8), 7.85 (t, 1H, *J*=7.8, H-9); ^13^C NMR (125 MHz) δ: 162.70 (C-2), 142.92 (C-6a), 139.51 (C-10b), 134.32 (C-5), 132.98 (C-3 Ph.), 132.97 (C-8), 132.34 (C-1 Ph.), 131.71 (C-5 Ph.), 131.04 (C-2 Ph.), 129.71 (C-4 Ph.), 129.08 (C-7), 127.02 (C-9), 126.09 (C-6 Ph.), 123.91 (C-10), 113.36 (C-10a); LC-MS, *m/z* = 281 [M+1], 283 [M+3]; Anal. calcd. for C_15_H_9_ClN_4_: C, 64.18; H, 3.23; Cl, 12.63; N, 19.96; Found: C, 64.21; H, 3.26; Cl, 12.64; N, 19.97.

#### 2-(4-Chlorophenyl)[1,2,4]triazolo[1,5-c]quinazoline (**5.25**)

Yield: Method A, 92%; Method B, 70.5%; M.p. >300°C (dioxane–H_2_O); LC-MS, *m/z* = 281 [M+1], 283 [M+3]; Anal. calcd. for C_15_H_9_ClN_4_: C, 64.18; H, 3.23; Cl, 12.63; N, 19.96; Found: C, 64.19; H, 3.24; Cl, 12.62; N, 19.98.

#### 2-(2-Bromophenyl)[1,2,4]triazolo[1,5-c]quinazoline (**5.26**)

Yield: Method A, 90%; M.p. 162–164°C (dioxane); EI-MS, *m/z* (I_rel_, %) = 327 (4.4), 326 (93.5), 325 (M^+•^, 32.1), 324 (100), 323 (17.8), 245 (29.1), 198 (11.1), 196 (13.0), 190 (8.4), 149 (5.2), 143 (5.8), 129 (17.6), 117 (12.4), 116 (14.1), 115 (14.6), 114 (14.3), 108 (9.1), 103 (5.0), 102 (32.8), 90 (23.8), 89 (24.9), 88 (38.0), 87 (8/9), 76 (14.6), 75 (17.5), 65 (5.0), 64 (6.9), 63 (13.8), 62 (25.2), 51 (8.5); LC-MS, *m/z* = 327 [M+2], 328 [M+3]; Anal. calcd. for C_15_H_9_BrN_4_: C, 55.41; H, 2.79; Br, 24.57; N, 17.23; Found: C, 55.39; H, 2.76; Br, 24.56; N, 17.21.

#### 2-(4-Bromophenyl)[1,2,4]triazolo[1,5-c]quinazoline (**5.27**)

Yield: Method A, 92%; Method B, 81.2%; Method C, 53.6%; M.p. 296–298°C (DMF–H_2_O); EI-MS, *m/z* (I_rel_, %) = 327 (14.3), 326 (87.9), 325 (M^+•^, 38.0), 324 (86.2), 323 (27.5), 262 (4.9), 245 (4.6), 217 (3.7), 198 (8.9), 196 (9.3), 190 (7.4), 183 (3.8), 181 (4.5), 169 (11.3), 167 (12.1), 164 (3.1), 155 (3.3), 149 (4.2), 130 (4.3), 129 (42.0), 119 (3.5), 117 (9.1), 116 (14.1), 115 (14.3), 114 (15.3), 103 (9.1), 102 (89.4), 100 (4.0), 91 (5.2), 90 (34.1), 89 (25.4), 88 (100.0), 87 (33.9), 86 (14.0), 85 (4.2), 81 (3.2), 77 (6.1), 76 (29.4), 75 (34.2), 74 (10.4), 65 (3.5), 64 (6.3), 63 (17.5), 62 (35.3), 61 (5.1), 55 (3.9), 52 (5.9), 51 (16.7), 50 (17.3), 41 (3.3), 39 (9.6), 38 (3.4), 32 (6.1), 28 (10.0); LC-MS, *m/z* = 325 [M], 327 [M+2]; Anal. calcd. for C_15_H_9_BrN_4_: C, 55.41; H, 2.79; Br, 24.57; N, 17.23; Found: C, 55.43; H, 2.81; Br, 24.59; N, 17.25.

#### 2-(3-Nitrophenyl)[1,2,4]triazolo[1,5-c]quinazoline (**5.28**)

Yield: Method A, 44%; M.p. >300°C (DMF); LC-MS, *m/z* = 292 [M+1]; Anal. calcd. for C_15_H_9_N_5_O_2_: C, 61.85; H, 3.11; N, 24.04; Found: C, 61.87; H, 3.14; N, 24.06.

#### 2-(4-Nitrophenyl)[1,2,4]triazolo[1,5-c]quinazoline (**5.29**)

Yield: Method A, 78%; M.p. >300°C (DMF); LC-MS, *m/z* = 292 [M+1]; Anal. calcd. for C_15_H_9_N_5_O_2_: C, 61.85; H, 3.11; N, 24.04; Found: C, 61.86; H, 3.15; N, 24.07.

#### 2-(2-Furyl)[1,2,4]triazolo[1,5-c]quinazoline (**5.30**)

Yield: Method A, 66%; M.p. 162–166°C (dioxane–H_2_O); ^1^H-NMR (400 MHz) δ: 9.64 (s, 1H, H-5), 8.50 (d, 1H, *J* = 7.8, H-10), 8.09 (d, 1H, *J =* 7.8, H-7), 8.00 (s, 1H, H-4′), 7.96 (t, 1H, *J =* 7.8, H-9), 7.85 (t, 1H, *J =* 7.8, H-8), 7.33 (d, 1H, *J* = 3.1, H-3′), 6.77 (s, 1H, H-5′.); EI-MS, *m/z* (I_rel_, %) = 237 (51.3), 236 (M^+•^, 100), 235 (8.2), 207 (6.7), 129 (23.2), 114 (6.7), 109 (6.0), 108 (91.3), 104 (8.3), 103 (13.1), 102 (37.4), 80 (6.5), 79 (8.3), 76 (10.9), 75 (9.8), 71 (21.5), 52 (12.2), 51 (12.8), 50 (8.3), 43 (9.8), 41 (5.1); LC-MS, *m/z* = 237 [M+1]; Anal. calcd. for C_13_H_8_N_4_O: C, 66.10; H, 3.41; N, 23.72; Found: C, 66.13; H, 3.46; N, 23.75.

#### 2-(3-Furyl)[1,2,4]triazolo[1,5-c]quinazoline (**5.31**)

Yield: Method A, 73%; Method C, 23%; M.p. 152–156°C (dioxane-H_2_O); ^1^H-NMR (400 MHz) δ: 9.62 (s, 1H, H-5), 8.54 (s, 1H, H-4′), 8.49 (d, 1H, *J* = 7.9, H-10), 8.09 (d, 1H, *J* = 7.9, H-7), 7.97 (t, 1H, *J* = 7.9, H-9), 7.92 (s, 1H, H-2′), 7.85 (t, 1H, *J* = 7.9, H-8), 7.09 (s, 1H, H-5′); EI-MS, *m/z* (I_rel_, %) = 237 (16.2), 236 (M^+•^, 100), 235 (6.2), 208 (21.1), 207 (5.0), 180 (7.1), 179 (10.5), 154 (5.2), 153 (5.1), 130 (5.0), 129 (13.9), 115 (5.2), 102 (5.6), 88 (13.7), 62 (8.9), 51 (6.8); LC-MS, *m/z* = 237 [M+1]; Anal. calcd. for C_13_H_8_N_4_O: C, 66.10; H, 3.41; N, 23.72; Found: C, 66.09; H, 3.39; N, 23.69.

#### 2-(2-Thienyl)[1,2,4]triazolo[1,5-c]quinazoline (**5.32**)

Yield: Method A, 77%; M.p. 178–180°C (dioxane–H_2_O); ^1^H-NMR (400 MHz) δ: 9.64 (s, 1H, H-5), 8.52 (d, 1H, *J* = 7.9, H-10), 8.10 (d, 1H, *J* = 7.9, H-7), 8.01-7.94 (m, 2H, H-8, H-3′), 7.89-7.82 (m, 2H, H-9, H-5′), 7.31-7.28 (t, 1H, H-4′); ^13^C NMR (125 MHz) δ: 160.22 (C-2), 151.20 (C-6a), 142.98 (C-10b), 139.28 (C-5), 132.89 (C-2 Th.), 132.83 (C-8), 130.07 (C-5 Th.), 129.57 (C-4 Th.), 129.07 (C-3 Th.), 129.01 (C-7), 128.98 (C-9), 123.88 (C-10), 117.81 (C-10a);EI-MS, *m/z* (I_rel_, %) = 253 (14.9), 252 (M^+•^, 100), 251 (11.1), 129 (10.5), 124 (36.4), 102 (5.6), 97 (11.0), 96 (8.1), 95 (7.1), 88 (8.0), 69 (5.7), 62 (9.9); LC-MS, *m/z* = 253 [M+1]; Anal. calcd. for C_13_H_8_N_4_S: C, 61.89; H, 3.20; N, 22.21; S, 12.71; Found: C, 61.87; H, 3.21; N, 22.22,; S, 12.73.

#### 2-(3-Thienyl)[1,2,4]triazolo[1,5-c]quinazoline (**5.33**)

Yield: Method A, 51%; Method C, 51%; M.p. 164–166°C (dioxane–H_2_O); ^1^H-NMR (400 MHz) δ: 9.64 (s, 1H, H-5), 8.51 (d, 1H, *J* = 7.8, H-10), 8.40 (s, 1H, H-4′), 8.09 (d, 1H, *J* = 7.8, H-7), 7.97 (t, 1H, *J* = 7.8, H-9), 7.86 (t, 1H, *J* = 7.8, H-8), 7.79 (s, 2H, H-2′, H-5′); EI-MS, *m/z* (I_rel_, %) = 254 (6.9), 253 (21.4), 252 (M^+•^, 100), 251 (23.9), 226 (5.4), 129 (6.8), 102 (5.9); LC-MS, *m/z* = 253 [M+1]; Anal. calcd. for C_13_H_8_N_4_S: C, 61.89; H, 3.20; N, 22.21; S, 12.71; Found: C, 61.88; H, 3.23; N, 22.24,; S, 12.75.

#### 2-(1H-Pyrrol-2-yl)[1,2,4]triazolo[1,5-c]quinazoline (**5.34**)

Yield: Method A, 65%; M.p. 248–250°C (dioxane–H_2_O); ^13^C NMR (125 MHz) δ: 159.47 (C-2), 150.90 (C-6a), 143.02 (C-10b), 139.21 (C-5), 132.63 (C-8), 129.39 (C-7), 128.98 (C-9), 123.73 (C-10), 122.51 (C-5 Pyr.), 122.38 (C-2 Pyr.), 117.85 (C-3 Pyr.), 111.08 (C-10a), 109.96 (C-4 Pyr.); EI-MS, *m/z* (I_rel_, %) = 236 (16.0), 235 (M^+•^, 100), 129 (6.9), 115 (6.4), 107 (18.8), 102 (7.6), 92 (5.7); 80 (11.6), 79 (7.0), LC-MS, *m/z* = 237 [M+2]; Anal. calcd. for C_13_H_9_N_5_: C, 66.37; H, 3.86; N, 29.77; Found: C, 66.35; H, 3.86; N, 29.76.

#### Ethyl 2,4-dimethyl-5-([1,2,4]triazolo[1,5-c]quinazolin-2-yl)-1H-pyrrole-3-carboxylate (**5.35**)

Yield: Method A, 73%; M.p. 190–192°C (dioxane–H_2_O); ^1^H-NMR (400 MHz) δ: 12.06 (s, 1H, -NH), 9.61 (s, 1H, H-5), 8.50 (d, 1H, *J* = 8.0, H-10), 8.10 (d, 1H, *J* = 8.0, H-7), 7.97 (t, 1H, *J* = 8.0, H-9), 7.86 (t, 1H, *J* = 8.0, H-8), 4.22 (q, 2H, *J* = 7.1 Гц, -CH_2_), 2.71 (s, 3H, 2-CH_3_), 2.51 (s, 3H, 4-CH_3_), 1.31 (t, 3H, *J* = 7.1, -CH_3_); EI-MS, *m/z* (I_rel_, %) = 336 (14.2), 335 (M^+•^, 100), 307 (9.0), 306 (45.7), 291 (12.3), 290 (50.0), 289 (34.9), 288 (30.2), 263 (14.3), 262 (29.5), 261 (67.8), 260 (19.1), 196 (12.4), 147 (6.2), 145 (12.4), 144 (6.9), 118 (6.3), 117 (7.5), 88 (5.0), 77 (5.4); LC-MS, *m/z* = 336 [M+1]; Anal. calcd. for C_18_H_17_N_5_O_2_: C, 64.47; H, 5.11; N, 20.88; Found: C, 64.46; H, 5.09; N, 20.86.

#### 2-(1-Benzofuran-2-yl)[1,2,4]triazolo[1,5-c]quinazoline (**5.36**)

Yield: Method A, 94%; M.p. 262–264°C (dioxane–H_2_O); EI-MS, *m/z* (I_rel_, %) = 287 (19.3), 286 (M^+•^, 100), 158 (10.1), 143 (5.3), 129 (9.4), 115 (8.1), 103 (13.8), 102 (11.1), 88 (8.1), ; LC-MS, *m/z* = 287 [M+1]; Anal. calcd. for C_17_H_10_N_4_O: C, 71.32; H, 3.52; N, 19.57; Found: C, 71.31; H, 3.49; N, 19.55.

#### 2-(1-Benzothiophen-2-yl)[1,2,4]triazolo[1,5-c]quinazoline (**5.37**)

Yield: Method A, 54%; M.p. 216–218°C (dioxane–H_2_O); ^1^H-NMR (400 MHz) δ: 9.69 (s, 1H, H-5), 8.54 (d, 1H, *J* = 7.5, H-10), 8.35 (s, 1H, H-3′), 8.16-8.02 (m, 3H, H-7, H-4′, H-7′), 7.99 (t, 1H, *J* = 7.5, H-9), 7.89 (t, 1H, *J* = 7.5, H-8), 7.53-7.45 (m, 2H, *J* = 7.6, H-5′, H-6′); ^13^C NMR (125 MHz) δ: 160.16 (C-2), 151.44 (C-6a), 143.02 (C-7a Benzoth.), 140.35 (C-3a Benzoth.), 139.94 (C-10b), 139.40 (C-5), 133.06 (C-8), 133.05 (C-4 Benzoth.), 129.70 (C-7), 129.09 (C-9), 126.59 (C-6 Benzoth.), 126.05 (C-5 Benzoth.), 125.59 (C-3 Benzoth.), 125.36 (C-2 Benzoth.), 123.92 (C-10), 123.29 (C-7 Benzoth.), 117.88 (C-10a); EI-MS, *m/z* (I_rel_, %) = 304 (7.3), 303 (23.3), 302 (M^+•^, 100), 301 (5.7), 174 (6.7), 159 (7.0), 147 (5.0), 146 (5.5), 129 (5.0), 102 (6.3), 88 (5.1); LC-MS, *m/z* = 303 [M+1]; Anal. calcd. for C_17_H_10_N_4_S: C, 67.53; H, 3.33; N, 18.53; S, 10.61; Found: C, 67.54; H, 3.36; N, 18.56; S, 10.63.

#### 2-(1H-Indol-2-yl)[1,2,4]triazolo[1,5-c]quinazoline (**5.38**)

Yield: Method A, 60%; M.p. 278–280°C (dioxane–H_2_O); ^1^H-NMR (400 MHz) δ: 12.09 (s, 1H, -NH), 9.65 (s, 1H, H-5), 8.53 (d, 1H, *J* = 7.8, H-10), 8.11 (d, 1H, *J* = 7.8, H-7), 7.97 (t, 1H, *J* = 7.8, H-9), 7.87 (t, 1H, *J* = 7.8, H-8), 7.67 (d, 1H, *J* = 7.8, H-7′), 7.53 (d, 1H, *J* = 7.8, H-4′), 7.29 (s, 1H, H-3′), 7.21 (t, 1H, *J* = 7.8, H-6′), 7.08 (t, 1H, *J* = 7.8, H-5′); ^13^C NMR (125 MHz) δ: 159.20 (C-2), 151.21 (C-6a), 143.03 (C-10b), 139.36 (C-7a Ind.), 138.01 (C-5), 132.85 (C-8), 129.61 (C-3a Ind.), 129.07 (C-7), 128.61 (C-9), 128.29 (C-10), 123.80 (C-5 Ind.), 123.56 (C-4 Ind.), 121.47 (C-6 Ind.), 120.32 (C-2 Ind.), 117.91 (C-7 Ind.), 112.68 (C-10a), 103.89 (C-3 Ind.);EI-MS, *m/z* (I_rel_, %) = 286 (20.6), 285 (M^+•^, 100), 160 (12.7), 157 (5.3), 144 (29.8), 142 (7.0), 130 (5.4), 129 (9.0), 116 (5.9), 115 (6.1), 103 (8.4), 102 (5.9), 89 (11.6); LC-MS, *m/z* = 286 [M+1]; Anal. calcd. for C_17_H_11_N_5_: C, 71.57; H, 3.89; N, 24.55; Found: C, 71.59; H, 3.91; N, 25.57.

#### 2-(Pyridin-3-yl)[1,2,4]triazolo[1,5-c]quinazoline (**5.39**)

Yield: Method A, 90%; M.p. 210–212°C (2-propanol–H_2_O); ^1^H-NMR (400 MHz) δ: 9.70 (s, 1H, H-5), 9.41 (dd, 1H, ^4^*J* = 2.1, ^5^*J* = 0.8, H-2′), 8.75 (dd, 1H, ^3^*J* = 4.8, ^4^*J* = 1.7, H-6′), 8.59 (dt, 1H, ^3^*J* = 7.9, ^4^*J* = 1.9, H-4′), 8.55 (d, 1H, *J* = 8.0, H-10), 8.11 (d, 1H, *J* = 8.0, H-7), 7.95 (t, 1H, *J* = 8.0, H-8), 7.87 (t, 1H, H-9), 7.62 (m, 1H, H-5′); LC-MS, *m/z* = 248 [M+1]; Anal. calcd. for C_14_H_9_N_5_: C, 68.01; H, 3.67; N, 28.32; Found: C, 67.98; H, 3.64; N, 28.30.

#### 2-(Pyridin-4-yl)[1,2,4]triazolo[1,5-c]quinazoline (**5.40**)

Yield: Method B, 57%; M.p. 246–248°C (dioxane); ^1^H-NMR (400 MHz) δ: 9.72 (s, 1H, H-5), 8.83, 8.19 (d, 2H, *J* = 5.0, H-2′,3′,5′,6′), 8.56 (d, 1H, H-10), 8.12 (d, 1H, *J* = 8.0, H-7), 7.99 (t, 1H, *J* = 7.8, H-8), 7.88 (t, 1H, *J* = 7.8, H-9); LC-MS, *m/z* = 248 [M+1]; Anal. calcd. for C_14_H_9_N_5_: C, 68.01; H, 3.67; N, 28.32; Found: C, 68.03; H, 3.69; N, 28.34.

### General procedure for (3H-quinazoline-4-ylidene)hydrazide dicarboxylic acids (6.1–6.3)

*Method A.* (**6.1**) To a mixture of 1.6 g (10 mM) of 4-hydrazinoquinazoline (**1.1**) and 1.1 g (11 mM) of triethylamine in 10 ml of anhydrous dioxane, 11 mM of ethyl chlorooxalate was added dropwise while stirring in the water bath at 60–80°C for 10 min with a calcium chloride tube. Then the reaction mixture was left at room temperature for 8–12 h while stirring. The resulting mixture was poured into the water, and the formed precipitate was filtered and dried.

*Method B* (**6.2, 6.3**). To a mixture of 1.6 g (10 mM) of the 4-hydrazinoquinazoline (**1.1**) in 10 ml of anhydrous dioxane, 11 mM of the corresponding carboxylic acid (succinic or phthalic acid) anhydride was added and heated in the water bath at 60–80°C for 1–1.5 h. Then the reaction mixture was left at room temperature for 8–12 h. The resulting mixture was poured into the water, and the formed precipitate was filtered and dried.

#### Ethyl oxo[2-(quinazolin-4(3H)-ylidene)hydrazino]acetate (**6.1**)

Yield: Method A, 85%; M.p. 216–218°C (ethanol–H_2_O); ^1^H-NMR (400 MHz) δ: 12.78/12.04 (s, 1H, NH), 11.21 (s, 1H, NH), 8.79/8.07 (s, 1H, H-2), 8.19/7.95 (d, 1H, *J* = 7.8, H-5), 7.75-7.24 (m, 3H, H-6, 7, 8), 4.28/3.43 (q, 2H, *J* = 7.1, CH_2_), 1.30/1.05 (t, 3H, *J* = 7.1, CH_3_); LC-MS, *m/z* = 261 [M+1]; Anal. calcd. for C_12_H_12_N_4_O_3_: C, 55.38; H, 4.65; N, 21.53; Found: C, 55.35; H, 4.65; N, 21.54.

#### 4-Oxo-4-[2-(quinazolin-4(3H)-ylidene)hydrazino]butanoic acid (**6.2**)

Yield: Method B, 97%; M.p. 182–184°C (H_2_O); ^1^H-NMR (400 MHz) δ: 12.15/11.58 (s, 1H, NH), 10.21/9.58 (s, 1H, NH), 8.20-7.20 (m, 5H, H_apom_), 2.85 (m, 4H, CH_2_CH_2_); LC-MS, *m/z* = 261 [M+1]; Anal. calcd. for C_12_H_12_N_4_O_3_: C, 55.38; H, 4.65; N, 21.53; Found: C, 55.40; H, 4.68; N, 21.56.

#### 2-{[2-(Quinazolin-4(3H)-ylidene)hydrazino]carbonyl}benzoic acid (**6.3**)

Yield: Method B, 99%; M.p. 286–288°C (2-propanol–H_2_O); 1H-NMR (400 MHz) δ: 12.98/11.62 (s, 1H, NH), 10.29/9.90 (s, 1H, NH), 8.10-7.10 (m, 9H, H_arom_); LC-MS, *m/z* = 309 [M+1]; Anal. calcd. for C_16_H_12_N_4_O_3_: C, 62.33; H, 3.92; N, 18.17; Found: C, 62.35; H, 3.95; N, 18.18.

### General procedure for ([1,2,4]triazolo[1,5-c]quinazolin-2-yl)carboxylic acids (7.1–7.2)

To 10 mM of dicarboxylic acid (3*H*-quinazolines-4-ylidene)hydrazides (**6.1, 6.2**), 10 ml of glacial acetic acid was added and refluxed for 3–6 h. The solvent was distilled off and water or a water-alcohol mixture was added to the residue. The formed precipitate was filtered off and dried.

#### Ethyl [1,2,4]triazolo[1,5-c]quinazoline-2-carboxylate (**7.1**)

Yield: 67%; M.p. 184–186°C (ethanol); ^1^H-NMR (400 MHz) δ: 9.52 (s, 1H, H-5), 8.54 (d, 1H, *J* = 7.7, H-10), 8.05 (d, 1H, *J* = 7.7, H-7), 7.92 (t, 1H, *J* = 7.7, H-8), 7.83 (t, 1H, *J* = 7.7, H-9), 4.46 (q, 2H, *J* = 7.1, CH_2_), 1.44 (t, 3H, *J* = 7.2, CH_3_); ^13^C NMR (125 MHz) δ: 159.92 (C-2), 156.05 (C=O), 151.10 (C-6a), 142.72 (C-10b), 139.69 (C-5), 133.24 (C-8), 130.01 (C-7), 129.13 (C-9), 123.92 (C-10), 118.25 (C-10a), 62.37 (-*C*H_2_-CH_3_), 14.52 (-CH_2_-*C*H_3_); LC-MS, *m/z* = 243 [M+1]; Anal. calcd. for C_12_H_10_N_4_O_2_: C, 59.50; H, 4.16; N, 23.13; Found: C, 59.53; H, 4.19; N, 23.16.

#### 3-([1,2,4]Triazolo[1,5-c]quinazolin-2-yl)propanoic acid (**7.2**)

Yield: 56%; M.p. 198–200°C (ethanol); ^1^H-NMR (400 MHz) δ: 11.90 (s, 1H, COOH), 9.26 (s, 1H, H-5), 8.42 (d, 1H, *J* = 7.7, H-10), 7.98 (d, 1H, *J* = 7.7, H-7), 7.82 (t, 1H, *J* = 7.6, H-8), 7.71 (t, 1H, *J* = 7.6, H-9), 3.18 (t, 2H, *J* = 7.2, CH_2_C*H*_2_), 2.82 (t, 2H, *J* = 7.3, C*H*_2_CH_2_); EI-MS, *m/z* (I_rel_, %) = 242 (M^+·^, 5.7), 225 (9.5), 198 (20.9), 197 (100), 196 (11.4), 129 (11.3), 102 (7.6), 75 (14.3); LC-MS, *m/z* = 243 [M+1]; Anal. calcd. for C_12_H_10_N_4_O_2_: C, 59.50; H, 4.16; N, 23.13; Found: C, 59.49; H, 4.13; N, 23.12.

### 2-[Quinazolin-4(3H)-ylidenamino]-1H-isoindole-1,3(2H)-dione (7.3)

To 8.3 g (10 mM) of the 2-{[2-quinazolin-4(3*H*)-ylidenhydrazino]carbonyl}benzoic acid (**6.3**), 10 ml of glacial acetic acid was added and refluxed for 3–6 h. The solvent was distilled off and water or a water-alcohol mixture was added to the residue. The formed precipitate was filtered off and dried.

Yield: 70.8%; M.p. 288–290°C (dioxane–H_2_O); ^1^H-NMR (400 MHz) δ: 12.03 (s, 1H, NH), 8.39 (m, 2H, H-2, 5), 8.20-7.60 (m, 7H, H_arom_); ^13^C-NMR (100 MHz) δ: 166.18 (1, 3), 157.94 (8a′), 156.29 (4′), 134.94 (7′), 134.15 (2′), 133.26 (5, 6), 132.07 (3a, 7a), 126.44 (5), 125.95 (6), 125.37 (4′a), 122.16 (8), 118.19 (4, 7); EI-MS, *m/z* (I_rel_, %) = 291 (8.8), 290 (M^+·^, 49.5), 246 (18.3), 245 (100), 207 (7.6), 147 (5.6), 129 (12.4), 118 (11.0), 104 (30.6), 103 (15.2), 102 (18.1), 90 (6.4), 77 (8.8), 76 (30.5), 75 (10.5), 50 (10.6), 44 (20.9); LC-MS, *m/z* = 291 [M+1]; Anal. calcd. for C_16_H_10_N_4_O_2_: C, 66.20; H, 3.47; N, 19.30; Found: C, 66.23; H, 3.49; N, 19.33.

### Biological assay

#### Cytotoxic activity against malignant human tumor cells

The primary anticancer assay was performed against a human tumor cell lines panel derived from nine neoplastic diseases, in accordance with the protocol of the Drug Evaluation Branch, National Cancer Institute, Bethesda [21–23]. The human tumor cell lines of the cancer screening panel were grown in RPMI 1640 medium containing 5% fetal bovine serum and 2 mM L-glutamine. For a typical screening experiment, cells were inoculated in 96 well microtiter plates in 100 mL assay volume, at plating densities ranging from 5000 to 40000 cell/well. After cell inoculation, the microtiter plates were incubated at 37°C, under an atmosphere of 5:95 CO_2_:air (v/v) at 100% relative humidity, for 24 h prior to the addition of drugs under assessment. Following drug addition (1 μM), the plates were incubated for an additional 48 h, under the same conditions. Sulforhodamine B (SRB) solution (100 μL, 0–4% w/v in 1% aq. acetic acid) was added to each well and plates were incubated for 10 min at room temperature. End point determinations were made with the protein binding dye, SRB. Results for each tested compound were reported as the percent of growth of the treated cells when compared to the untreated control cells. The cytotoxic and/or growth inhibitory effects of the most active selected compounds were tested *in vitro* against the full panel of about 60 human tumor cell lines at 10-fold dilutions of five concentrations ranging from 10^−4^ to 10^−8^ M. A 48-h continuous drug exposure protocol was followed and an SRB protein assay was used to estimate cell viability or growth. Using the seven absorbance measurements [time zero, (T_z_), control growth in the absence of drug (C), and test growth in the presence of drug at the five concentration levels (T_i_)], the percentage growth was calculated at each of the drug concentration levels. Percentage growth inhibition was calculated as:

$$\left[\left({\text{T}}_{\text{i}}-{\text{T}}_{\text{z}}\right)/\left(\text{C}-{\text{T}}_{\text{z}}\right)\right]\times 100\;\text{for concentrations for which }{\text{T}}_{\text{i}}\geq {\text{T}}_{\text{z}},$$

$$\left[\left({\text{T}}_{\text{i}}-{\text{T}}_{\text{z}}\right)/{\text{T}}_{\text{z}}\right]\times 100\;\text{for concentrations for which }{\text{T}}_{\text{i}}{<\text{T}}_{\text{z}}.$$

Three dose-response parameters were calculated for each compound. Growth inhibition of 50% (GI_50_) was calculated from [(T_i_ − T^z^)/(C − T_z_)] × 100 = 50, which is the drug concentration resulting in a 50% lower net protein increase in the treated cells (measured by SRB staining) as compared to the net protein increase seen in the control cells. The drug concentration resulting in total growth inhibition (TGI) was calculated from T_i_ = T_z_. The LC_50_ (concentration of drug resulting in a 50% reduction in the measured protein at the end of the drug treatment as compared to that at the beginning) indicating a net loss of cells following treatment was calculated from [(T_i_ − T_z_)/T_z_] ×100 = −50. Values were calculated for each of these three parameters if the level of activity was reached; however, if the effect was not reached or was exceeded, the value for that parameter was expressed as greater or less than the maximum or minimum concentration tested. The log GI_50_, log TGI, log LC_50_ were then determined, defined as the mean of the log’s of the individual GI_50_, TGI, LC_50_ values. The lowest values were obtained with the most sensitive cell lines.

## Figures and Tables

**Sch. 1.**
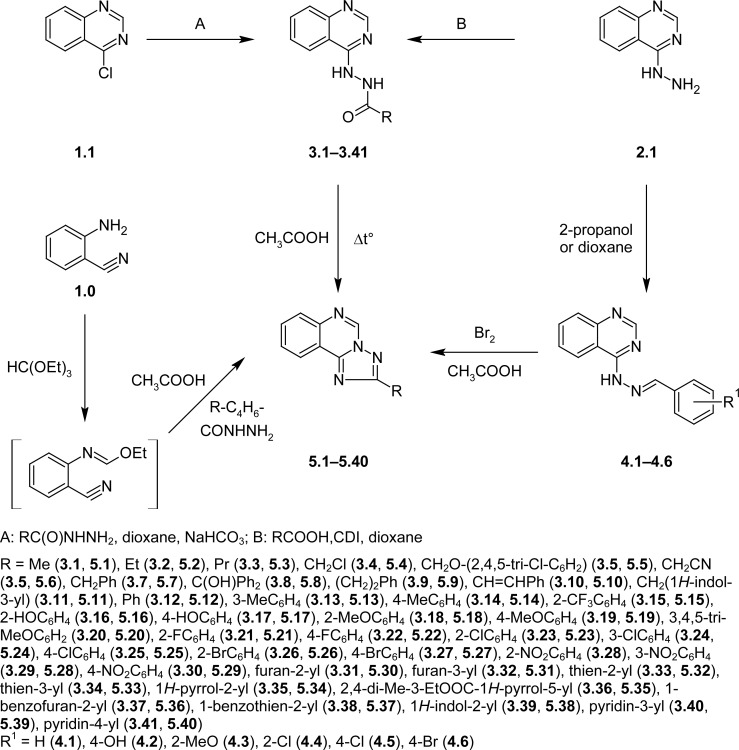
Synthesis of the carboxylic acid (3*H*-quinazolin-4-ylidene)hydrazides (**3.1–3.41**), *N*-(R-benzylidene)-*N′*-(3*H*-quinazolin-4-ylidene)hydrazones (**4.1–4.6**), and 2-R-[1,2,4]triazolo[1,5-*c*]quinazolines (**5.1–5.40**).

**Sch. 2.**
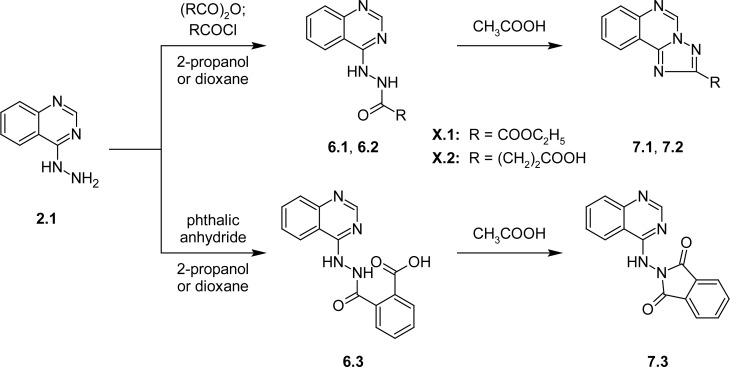
Synthesis of the dicarboxylic acid (3*H*-quinazolines-4-ylidene)hydrazides (**6.1–6.3**), ([1,2,4]triazolo[1,5-*c*]quinazolin-2-yl)carboxylic acids (**7.1–7.2**), and 2-[quinazolin-4(3*H*)-ylidenamino]-1*H*-isoindole-1,3(2*H*)-dione (**7.3**).

**Tab. 1.** Percentage of *in vitro* tumor cell lines growth at 10 μM for compounds

**Cpd.**	**Mean growth, %**		**Range of growth, %**	Cell line growth, %*
3.3	98.90		22.90–163.45	22.90 (IGROV1/OV), 67.22 (UO-31/RC)
3.6	96.94		54.03–139.67	69.83 (NCI-H322M/nscLC), 67.24 (RPMI-822/L), 67.69 (UO-31/RC), 54.03 (UACC-257/M)
3.10	86.93		1.02–169.63	56.77 (A549/ATCC/nscLC), 29.03 (HOP-92/nscLC), 1.02 (HS 578T/BC), 65.03 (MCF7/BC), 68.82 (MDA- MB-435/BC), 66.85 (IGROV1/OV), 66.91 (CCRF- CEM/L), 31.76 (RXF 393/RC)
3.19	96.26		34.30–151.07	34.30 (IGROV1/OV), 51.53 (RPMI-8226/L)
3.20	30.8		−22.19–76.81	34.45 (A549/ATCC/nscLC), 47.80 (EKVX/nscLC), 31.79 (HOP-62/nscLC), −20.29 (HOP-92/nscLC), 53.15 (NCI-H226/nscLC), 56.08 (NCI-H23/nscLC), 49.63 (NCI-H322M/nscLC), 6.93 (NCI-H460/nscLC), 18.28 (NCI-H522/nscLC), 48.35 (COLO 205/ColC), 28.33 (HCT-116/ColC), 37.98 (HCT-15/ColC), 12.96 (HT29/ColC), 28.18 (KM12/ColC), 18.19 (SW-620/ColC), 52.47 (BT-549/BC), −8.25 (HS 578T/BC), 0.99 (MCF7/BC), 54.69 (MDA-MB-231/ATCC/BC), −8.47 (MDA-MB-435/BC), 66.42 (NCI/ADR-RES/BC), 50.41 (T-47D/BC), 43.70 (IGROV1/OV), 8.02 (OVCAR-3/OV), 52.75 (OVCAR-4/OV), 24.28 (OVCAR-8/OV), 59.20 (SK-OV-3/OV), 16.34 (CCRF-CEM/L), −22.19 (HL-60(TB)/L), 13.53 (K-562/L), 30.40 (MOLT-4/L), 24.50 (RPMI-8226/L), 6.80 (SR/L), 34.92 (786-0/RC), −8.58 (A498/RC), 39.91 (ACHN/RC), 28.32 (CAKI-1/RC), 15.38 (RXF 393/RC), 56.09 (SN12C/RC), 59.47 (TK-10/RC), 54.96 (UO-31/RC), 42.29 (LOX IMVI/M), 40.18 (M14/M), 18.32 (MALME-3M/M), 63.78 (SK-MEL-2/M), 59.92 (SK-M EL-28/M), −9.71 (SK-MEL-5/M), 33.49 (UACC-257/M), 49.90 (UACC-62/M), 40.89 (DU-145/PC), 44.69 (SF-268/CNSC), −21.73 (SF-295/CNSC), 16.51 (SF-539/CNSC), 44.03 (SNB-19/CNSC), 27.05 (SNB-75/CNSC),
3.23	99.41		12.34–270.61	66.48 (HOP-92/nscLC), 12.34 (MCF7/BC), 52.14 (T-47D/BC)
3.26	89.40		0.00–127.72	45.71 (MCF7/BC), 0.00 (MDA-MB-46/BC), 46.22 (T-47D/BC), 52.80 (RPMI-8226/L), 68.89 (UO-31/RC), 59.42 (MALME-3M/M)
3.28	98.79		8.10–139.88	61.82 (HOP-92/nscLC), 8.10 (HS 578T/BC), 64.60 (MCF7/BC)
3.29	97.38		4.74–166.79	69.55 (A549/ATCC/nscLC), 44.70 (HOP-92/nscLC), 4.74 (HS 578T/BC), 47.90 (MCF7/BC), 69.97 (MDA-MB-435/BC), 43.34 (RXF 393/RC)
4.3		85.09	35.40–131.32	68.23 (NCI-H322M/nscLC), 63.71 (NCI-H522/nscLC), 63.23 (HCT-15/ColC), 52.82 (MCF7/BC), 35.40 (MDA-MB-468/BC), 54.36 (IGROV1/OV), 36.55 (OVCAR-4/OV), 67.16 (SK-OV-3/OV), 69.11 (CCRF-CEM/L), 66.47 (UO-31/RC), 67.87 (LOX IMVI/M)
4.4		80.63	11.11–133.38	64.62 (EKVX/nscLC), 57.47 (NCI-H226/nscLC), 54.10 (NCI-H322M/nscLC), 68.42 (NCI-H522/nscLC), 60.8 (HCT-15/ColC), 67.60 (BT-549/BC), 58.96 (MCF7/BC), 56.76 (MDA-MB-231/ATCC/BC), 11.11 (MDA-MB-468/BC), 35.06 (T-47D/BC), 56.00 (IGROV1/OV), 33.96 (OVCAR-4/OV), 64.8 (OVCAR-8/OV), 60.46 (SK-OV-3/OV), 64.24 (CCRF-CEM/L), 65.00 (RPMI-8226/L), 45.7 (A498/RC), 61.21 (UO-31/RC), 62.24 (SNB-75/CNSC)
5.1		104.30	56.82–383.66	56.82 (HOP-92/nscLC)
5.4		95.68	−11.68–179.79	69.57 (HOP-92/nscLC), −11.68 (HS 578T/BC), 69.04 (SK-MEL-5/M)
5.5		104.67	−20.21–144.50	−20.21 (HS 578T/BC)
5.6		96.00	27.55–129.58	27.55 (EKVX/nscLC), 70.49 (HOP-62/nscLC), 64.12 (RPMI-822/L), 70.32 (SR/L), 65.90 (UO-31/RC)
5.7		103.54	−1.55–200.25	−1.55 (HS 578T/BC)
5.8		104.48	36.90–362.61	55.71 (KM12/ColC), 36.90 (MALME-3M/M)
5.10		57.02	−10.11–120.31	25.07 (A549/ATCC/nscLC), 56.27 (HOP-62/nscLC), 11.95 (HOP-92/nscLC), 58.89 (NCI-H23/nscLC), 20.32 (NCI-H460/nscLC), 40.58 (NCI-H522/nscLC), 44.03 (HCT-116/ColC), 43.63 (HCT-15/ColC), 47.67 (HT29/ColC), 42.78 (KM12/ColC), 32.84 (SW-620/ColC), 58.28 (BT-549/BC), 29.74 (MCF7/BC), 64.37 (MDA-MB-231/ATCC/BC), −10.11 (MDA-MB-435/BC), 21.67 (NCI/ADR-RES/BC), 46.30 (IGROV1/OV), 34.97 (OVCAR-3/OV), 23.63 (OVCAR-8/OV), 64.14 (CCRF-CEM/L), 59.79 (HL-60(TB)/L), 30.84 (K-562/L), 47.76 (RPMI-822/L), 14.20 (SR/L), 66.99 (786-0/RC), 44.33 (CAKI-1/RC), 10.54 (RXF 393/RC), 65.31 (UO-31/RC), 57.67 (LOX IMVI/M), 55.63 (M14/M), 57.27 (SK-M EL-28/M), 28.78 (SK-MEL-5/M), 60.50 (UACC-257/M), 65.67 (UACC-62/M), 58.40 (SF-268/CNSC), 29.74 (SF-295/CNSC), 65.45 (SF-539/CNSC), 67.66 (SNB-19/CNSC), 41.59 (SNB-75/CNSC)
5.11		92.80	54.29–123.87	54.29 (OVCAR-8/OV), 69.36 (RXF 393/RC)
5.12		92.78	39.61–134.95	58.18 (A549/ATCC/nscLC), 59.71 (EKVX/nscLC), 39.61 (HOP-92/nscLC), 64.01 (MCF7/BC), 51.34 (OVCAR-8/OV), 47.79 (RXF 393/RC)
5.14		104.63	73.65–137.04	73.65 (IGROV1/OV)
5.16		95.56	27.75–249.30	55.42 (EKVX/nscLC), 27.75 (HOP-92/nscLC), 66.42 (MCF7/BC), 66.10 (IGROV1/OV), 37.11 (OVCAR-3/OV), 65.38 (CAKI-1/RC), 68.03 (UO-31/RC)
5.17		97.03	37.23–160.30	58.87 (EKVX/nscLC), 47.50 (MCF7/BC), 57.10 (T-47D/BC), 37.23 (IGROV1/OV), 69.77 (TK-10/RC), 65.31 (UO-31/RC)
5.19		93.08	57.30–141.64	63.90 (RPMI-8226/L), 57.30 (UO-31/RC), 65.19 (T-47D/BC),
5.20		100.66	−13.36–157.85	−13.36 (HOP-92/nscLC), 53.13 (MCF7/BC)
5.29		96.03	−3.02–138.38	44.55 (HOP-92/nscLC), −3.02 (HS 578T/BC), 66.97 (MCF7/BC), 68.75 (RXF 393/RC), 45.45 (SF-295/CNSC)
5.30		94.13	−17.63–119.47	−17.63 (CCRF-CEM/L), 27.26 (MCF7/BC), 60.84 (T-47D/BC), −10.43 (MDA-MB-468/BC)
5.31		88.71	−11.11–129.80	24.74 (CCRF-CEM/L), 67.86 (SR/L), 43.06 (EKVX/nscLC), 61.45 (OVCAR-4/OV), 9.65 (MCF7/BC), 66.96 (MDA-MB-231/ATCC/BC), 48.02 (T-47D/BC), −11.11 (MDA-MB-468/BC)
5.32		94.59	59.59–136.55	66.18 (IGROV1/OV), 59.59 (UO-31/RC), 68.94 (MCF7/BC)
5.33		99.01	26.18–125.72	26.18 (CCRF-CEM/L), 68.08 (UO-31/RC)
5.34		106.35	70.04–169.09	70.04 (IGROV1/OV)
5.35		90.61	50.97–123.54	57.98 (MOLT-4/L), 50.97 (SK-MEL-5/M), 66.37 (UO-31/RC), 61.83 (T-47D/BC),
5.36		90.12	59.31–126.37	60.03 (NCI-H460/nscLC), 67.92 (COLO 205/ColC), 59.31 (SNB-75/CNSC), 60.51 (IGROV1/OV), 62.04 (ACHN/RC), 61.28 (UO-31/RC)
5.37		94.59	50.08–140.25	64.75 (SNB-75/CNSC), 59.99 (IGROV1/OV), 50.08 (UO-31/RC), 68.73 (MCF7/BC)
5.38		85.80	20.42–114.33	20.42 (CCRF-CEM/L), 65.58 (EKVX/nscLC), 66.61 (NCI-H522/nscLC), 62.76 (HCT-15/ColC), 64.49 (KM12/ColC), 31.00 (MDA-MB-435/M), 65.11 (MDA-MB-231/ATCC/BC)
5.39		97.63	−13.96–127.11	−13.96 (HS 578T/BC), 59.37 (MCF7/BC)
5.40		95.31	36.81–137.76	56.22 (HOP-92/nscLC), 53.37 (NCI-H322M/nscLC), 63.59 (KM12/ColC), 62.95 (OVCAR-8/OV), 59.32 (TK-10/RC)
6.1		102.41	−15.21–132.72	−15.21 (HS 578T/BC)
6.2		106.5	31.06–153.54	31.06 (IGROV1/OV)
6.3		101.59	−5.08–129.92	−5.08 (HS 578T/BC)
7.1		104.40	38.46–255.43	38.46 (HOP-92/nscLC), 62.58 (OVCAR-3/OV)
7.2		96.84	45.68–223.16	45.68 (HOP-92/nscLC), 67.82 (IGROV1/OV), 58.27 (MALME-3M/M), 69.40 (SK-MEL-5/M)
7.3		97.42	−6.36–170.82	65.12 (A549/ATCC/nscLC), 46.32 (HOP-92/nscLC), −6.36 (HS 578T/BC), 34.72 (RXF 393/RC)

* L – leukemia, nscLC – non-small cell lung cancer, ColC – colon cancer, CNSC – CNS cancer, M – melanoma, OV– ovarian cancer, RC – renal cancer, PC – prostate cancer, BC – breast cancer.

**Tab. 2.** The influence of compounds on the growth of individual tumor cell lines (GI_50_ ≤1.00 μM)

**Disease**	**Cell line**	**Compounds / dose-dependent parameters**			

		**3.20**		**5.10**	

		**GI_50_**	**TGI**	**GI_50_**	**TGI**
MG_MID		2.29	39.80	14.10	93.3
Leukemia	CCRF-CEM	2.81	>100	>100	>100
	HL-60(TB)	1.57	5.77	26.60	>100
	K-562	0.41	>100	–	–
	MOLT-4	2.93	>100	>100	>100
	RPMI-8226	2.31	>100	1.32	84.20
	SR	0.29	2.62	>100	>100
NSC lung cancer	A549/ATCC	5.27	>100	9.86	>100
	EKVX	5.13	>100	>100	>100
	HOP-62	1.24	>100	0.03	28.40
	HOP-92	2.93	>100	4.69	>100
	NCI-H226	1.67	5.97	2.06	83.40
	NCI-H23	2.86	>100	>100	>100
	NCI-H322M	7.01	>100	>100	>100
	NCI-H460	3.27	30.2	6.41	>100
	NCI-H522	0.34	6.55	4.26	>100
Colon cancer	COLO 205	1.71	5.86	43.60	>100
	HCC-2998	3.86	>100	>100	>100
	HCT-116	1.65	>100	3.43	>100
	HCT-15	1.92	>100	0.83	>100
	HT29	2.25	46.30	7.87	>100
	KM12	1.15	15.00	2.53	>100
	SW-620	1.31	>100	1.99	>100
CNS cancer	SF-268	4.76	>100	71.40	>100
	SF-295	0.95	8.00	9.38	>100
	SF-539	1.78	5.61	57.20	>100
	SNB-19	4.11	>100	>100	>100
	SNB-75	1.09	6.85	1.34	94.00
	U251	3.42	>100	8.40	>100
Melanoma	LOX IMVI	28.10	>100	16.80	>100
	MALME-3M	2.78	>100	19.80	>100
	M14	1.60	>100	2.32	>100
	MDA-MB-435	0.31	1.18	0.71	33.30
	SK-MEL-2	5.36	>100	73.30	>100
	SK-MEL-28	2.37	>100	15.50	>100
	SK-MEL-5	0.74	12.60	6.24	>100
	UACC-257	5.67	>100	>100	>100
	UACC-62	0.32	12.50	1.90	86.20
Ovarian cancer	IGROV1	3.03	62.30	1.71	>100
	OVCAR-3	0.33	1.31	2.95	92.10
	OVCAR-4	7.37	>100	>100	>100
	OVCAR-5	15.50	>100	>100	>100
	OVCAR-8	3.97	>100	35.00	>100
	NCI/ADR-RES	6.38	>100	34.30	>100
	SK-OV-3	2.20	>100	>100	>100
Renal cancer	786-0	2.83	>100	4.81	>100
	A498	1.13	9.96	>100	>100
	ACHN	5.80	>100	96.80	>100
	CAKI-1	4.81	>100	6.99	>100
	RXF 393	1.06	3.07	10.40	67.80
	TK-10	8.94	>100	>100	>100
	UO-31	6.21	>100	>100	>100
Prostate cancer	PC-3	0.56	>100	6.91	>100
	DU-145	1.91	4.52	>100	>100
Breast cancer	MCF7	0.52	>100	6.05	>100
	MDA-MB-231/ATCC	4.41	>100	0.34	>100
	HS 578T	1.67	6.01	6.21	89.00
	BT-549	40.40	>100	39.90	>100
	T-47D	2.49	>100	>100	>100
